# Contact tracing strategies for infectious diseases: A systematic literature review

**DOI:** 10.1371/journal.pgph.0004579

**Published:** 2025-05-09

**Authors:** Danielle Guy, Petya Kodjamanova, Lena Woldmann, Jyoti Sahota, Melanie Bannister-Tyrrell, Yajna Elouard, Marie-Amélie Degail

**Affiliations:** 1 Amaris, Health Economics and Market Access, Barcelona, Spain; 2 Amaris, Health Economics and Market Access, London, United Kingdom; 3 Amaris, Health Economics and Market Access, Toronto, Canada; 4 Nossal Institute for Global Health, University of Melbourne, Melbourne, Australia; 5 World Health Organization, Geneva, Switzerland; Federal University Birnin Kebbi, NIGERIA

## Abstract

Contact tracing has been a crucial public health strategy for breaking infectious diseases chains of transmission. Although many resources exist for disease outbreak management none address the rationale of contact tracing. This comprehensive review aims to evaluate contact tracing strategies, their effectiveness, and health systems governance across various diseases to inform a disease-agnostic contact tracing guideline. This systematic review was registered with PROSPERO (ID: CRD42023474507) and follows Preferred Reporting Items for Systematic reviews and Meta-Analyses (PRISMA) guidelines. Descriptive and interventional studies in the six official United Nations languages were included, excluding modelling studies and animal-to-human transmission. An electronic search was conducted in Embase, Medline, Medline-in-process, and Cochrane libraries from inception to September 2023. The revised Cochrane Risk of Bias Tool and the Risk of Bias in Non-Randomized Studies of Interventions were used for bias assessment. The search yielded 378 studies, primarily from Europe (29.6%) and North America (21.6%) and focusing on diseases such as the coronavirus disease (COVID-19) (47.4%) or tuberculosis (26.7%). 244 (64.5%) studies addressed contact tracing definitions, commonly based on physical proximity, including duration of contact and sexual partnerships (47.6%) and household exposure (27%). Effectiveness was examined in 330 (87.3%) studies, showing variation across diseases and contexts, with only five studies evaluating epidemiological impacts. Socio-cultural aspects were covered in 166 (43.9%) studies, revealing that stigma and public trust may affect the adherence to contact tracing. Health systems governance was discussed in 278 (73.5%) studies, emphasising the need for coordination among international organisations, national governments, and local health authorities, alongside a sustained and adequately supported workforce. This review provides critical insights into optimising contact tracing strategies. Effective contact tracing requires robust health systems governance, adequate resources, and community involvement. Future research should focus on establishing standardised metrics for comparative analysis and investigating the impact of contact tracing on disease incidence and mortality.

## Introduction

### Rationale

Contact tracing has been a longstanding public health strategy to break infectious disease chains of transmission. It has been documented as early as in the 17^th^ century for bubonic plague and was first operationalised in the beginning of the 20^th^ century to fight syphilis outbreaks [1–3]. Contact tracing continues to be used today to halt the spread of high priority epidemic-prone diseases including Ebola virus disease, measles, among others [4]. The unprecedented global response to the coronavirus disease (COVID-19) pandemic highlighted the need for internationally agreed upon standards for contact tracing [5].

The scale and volume of cases and contact persons during an epidemic or pandemic can overwhelm healthcare systems and public health agencies. The sheer magnitude of identifying, contacting, and monitoring many individuals necessitates substantial resources and coordination, often across varying levels of governments [6]. Additionally, the management of sensitive health data introduces challenges in terms of collecting and storing personal information while maintaining privacy and security, especially with the use of digital tools and databases [6].

Successful contact tracing heavily relies on community engagement, which can be hindered by consequences associated with being identified as a contact person and the interpretation and consequences of the use of contact tracing information [6–9]. Furthermore, the evolving nature of infectious diseases may require rapid adaptation of contact tracing protocols to address new variants and changing transmission dynamics [6]. Ethical considerations play a crucial role in balancing public health protection with individual rights and privacy, while efficiently and optimally deploying resources is essential for effective contact tracing efforts [6].

Despite its importance in controlling the spread of infectious diseases, contact tracing strategies are not without potential harms. Concerns include infringement on personal privacy and data security, stigmatisation and discrimination of individuals, risks associated with inaccurate results, and the resource-intensive nature of contact tracing [10–13].

Previous systematic reviews have assessed contact tracing for specific diseases or geographical contexts [1,6,14–23]. With evolving guidelines, technological advancements, and cultural variations, understanding optimal strategies remains a global public health challenge. Notably, key evidence gaps persist regarding the most effective design and implementation methods. Thus, a comprehensive review of contact tracing strategies and their effectiveness across diseases with diverse transmission modes and outbreak scenarios is essential for enhancing preparedness against endemic, epidemic, and emerging infectious diseases.

### Objectives

The absence of comprehensive global contact tracing strategy, guidelines and standard operating procedures was stressed during a consultation organised by the Global Outbreak Alert and response Network in June 2020 [24]. Although many resources exist for vertical program disease management, outbreak containment, and response none address the overall rationale of contact tracing and optimal strategies, nor give proper attention to the truly multisectoral nature of this public health intervention. This review was therefore commissioned by the World Health Organization (WHO) as part of the development of a disease-agnostic guideline on contact tracing primarily intended for national and subnational decision makers to develop guidance and implement actions to respond to outbreaks [25]. The review aimed to synthesise evidence on contact tracing strategies, regardless of disease area, offering insights into strategies, structures, impact measures, and resource requirements. As such, the review aspired to answer the following questions:

- What are the different contact tracing strategies used in controlling the spread of infectious diseases?
- How is effectiveness for contact tracing strategies defined and measured?
  How do epidemiological, socio-cultural, economic, and system factors influence the effectiveness of different types of contact tracing strategies?
  How are contact tracing strategies governed with respect to policy, ethics, stakeholder involvement, resource allocation (human and financial) and data protection?

## Methods

This review was registered with PROSPERO (ID: CRD42023474507) and adheres to Preferred Reporting Items for Systematic reviews and Meta-Analyses (PRISMA) guidelines [26]. The search was conducted on 14/09/2023.

### Eligibility criteria

Relevant descriptive and interventional studies reported in any of the six official United Nations languages, Arabic, Chinese, English, French, Russian, and Spanish, were included. No publication date restrictions were applied to the literature. Modelling studies were excluded due to uncertainties in assumptions and variability in model outputs. Contact tracing strategies for animal-to-human transmission also were excluded to focus on diseases with human-to-human transmission. Studies must have reported on contact tracing strategies or compared against another strategy. No grey literature was included.

### Information sources

An electronic database search was conducted in Embase, Medline, Medline-in-process, and Cochrane libraries from database inception to September 2023. Relevant conference proceedings and additional references from experts were also considered.

### Search strategy

The searches included a combination of keywords related to contact tracing and disease transmission (see S1 Table, S2 Table, S3 Table).

### Selection process

Records were downloaded into a bespoke database which was used to manage citation screening. After removal of duplicate citations, titles and abstracts of the retrieved citations were screened against the predefined inclusion and exclusion criteria (see S4 Table). Studies identified as potentially relevant based on their titles or abstracts were reviewed in full and selected for inclusion according to the same criteria. Articles were screened for inclusion by two reviewers in parallel and quality checked by another reviewer. Disagreements were resolved by discussion or if no agreement was reached a third reviewer was involved in the resolution.

### Data collection process

Data was extracted from all the included publications by four reviewers independently. Two additional independent reviewers conducted a quality check of the data extraction by reviewing 20% of the extractions.

### Data items

In addition to publication details and study characteristics, extracted items included disease, definitions of contact persons, features of contact tracing strategy, measures and size of effect, human and technical resource requirements, and community impacts (e.g., acceptability of contact tracing, adherence to contact tracing strategies, stigma surrounding contact tracing).

### Risk of bias assessment

The risk of bias assessment of randomized control trials outcomes was conducted using the revised Cochrane Risk of Bias Tool for Randomized Trials (RoB 2) [27]. The Risk Of Bias In Non-Randomized Studies - of Interventions (ROBINS-I) tool, recommended by the Cochrane Collaboration, was used for outcomes from non-randomised studies [28,29]. For qualitative studies, risk of bias was assessed using a framework based on the Critical Appraisal Skills Programme (CASP) assessment tool [30] that has been used in previous Cochrane Reviews [31–36].

### Certainty assessment

The certainty of evidence for key outcomes was assessed using Grading of Recommendations Assessment, Development and Evaluation (GRADE), including Confidence in the Evidence from Reviews of Qualitative Research (CerQual) for qualitative studies [37,38].

### Synthesis methods

The synthesis first involved tabulating results from included studies. Findings were then analysed descriptively across the four pre-defined focal areas relevant to public health decision making: contact tracing definitions, contact tracing effectiveness, socio-cultural aspects of contact tracing, and health systems governance of contact tracing strategies (i.e., processes, structures, and resources used to manage contact tracing) [39]. The narrative synthesis of contact tracing effectiveness focuses on non-digital contact tracing interventions as digital contact tracing interventions involve distinct methodologies and often have different primary outcomes.

### Patient and public involvement

It was not appropriate or possible to involve patients or the public in the design, or conduct, or reporting, or dissemination plans of our research.

## Results

**Fig 1:**
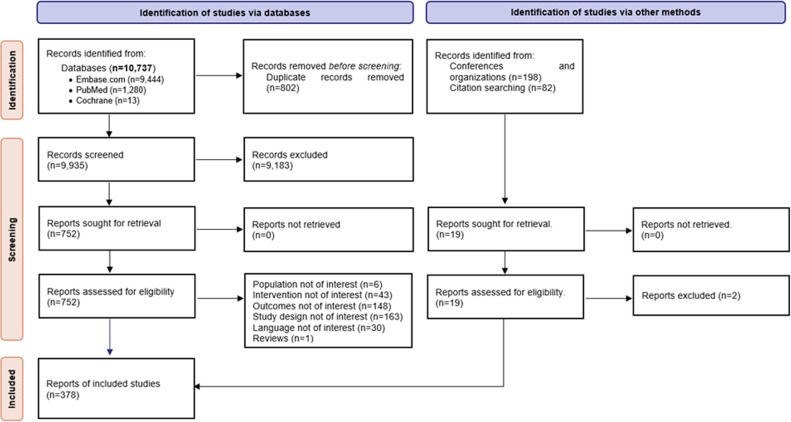
PRISMA Diagram.

In total, 378 studies met the selection criteria (see Fig 1).

### Study characteristics

By WHO region, 112 (29.6%) studies were from Europe, 82 (22.0%) from the Americas, 75 (19.8%) from the Western Pacific, 51 (13.5%) from Africa, 17 (4.5%) from South-East Asia, and 12 (3.2%) studies from the Eastern Mediterranean region. Most studies covered COVID-19 (n = 179; 47.4%) or tuberculosis (n = 101; 26.7%) and were retrospective (n = 91; 24.1%) or prospective (n = 79; 20.9%). There were only 12 (3.2%) randomized control trials. (see S1 Fig, S2 Fig).

### Risk of bias in included studies

Most randomized control trials (n = 9; 75.%) included had either some concerns (n = 5; 41.7.%) [40–44], or high risk of bias (n = 4; 33.3.%) [45–49]. Allocation concealment was a concern in some studies [40,42,48,49], while blinding was challenging due to the nature of contact tracing methods [43,48,49]. Most non-randomised studies (n = 21; 67.7%) were at critical or serious risk of bias [50–70]. This is primarily due to missing data as the true number of contact persons is likely unknown in any contact tracing strategy, as discussed in previous reviews on contact tracing [71]. For qualitative studies, the risk of bias assessment generally showed none to very minor concerns.

The detailed risk of bias assessments for all studies assessed are in S3 Fig, S4 Fig, S5 Table, S6 Table.

### Key definitions and elements of contact tracing strategies

#### Contact person definition.

Out of 378 included studies, 244 (64.5%) reported contact person definitions based on criteria such as physical proximity, including the duration of contact and sexual partnerships (47.6%), household exposure (27%), community exposure (14%), duration of exposure only (2%) or injection drug use (2%). Contact person definitions varied by disease type.

Most studies included used ‘close contact’ person and ‘high-risk contact’ person definitions interchangeably. However, two tuberculosis studies specifically defined high-risk contact persons with increased infection risk, such as immunocompromised individuals [72,73]. A detailed assessment is provided in S1 Box.

#### Contact person identification.

Contact person identification strategies were reported in 204 (54.0%) studies included. The main strategies are outlined in Table 1.

**Table 1:** Main contact person identification strategies described in included studies.

Main strategies to identify contact persons	Number of studies reporting type of contact person identification strategy	Examples
Contact tracing via phone interviews, online surveys, medical record review	102 (27.0%)	Interviewing cases or reviewing their medical records to ascertain numbers and types of potential contact persons [62,74,75].
Contact tracing via home visits and community investigation	70 (18.5%)	Contact tracers visit homes of index cases to identify close contact persons or household contact persons [76–81].
Use of apps and other technology	28 (7.4%)	Use of Bluetooth,[7,8,82–88] other real-time location services (RTLS) [51,57,58], or credit card transaction logs [89–92].
Contact tracing of the index case’s sex partners	28 (7.4%)	Contact tracers ask index cases to identify a minimum number of sexual contact persons within 90 days [93].
Contact tracing of the index case’s drug injection network	2 (0.5%)	Contact tracers asked the index case to list their partners freely until they could not recall any more partners [94,95].

#### Contact person follow-up and monitoring.

A total of 168 (44.4%) studies reported on contact person follow-up strategies. Contact person follow-up strategies were categorised as either ‘active follow-up’ or ‘passive follow-up’. Active follow-up strategies included those where the contact tracer actively follows-up and interacts with the contact person, passive follow-up included strategies without interaction between a contact tracer and the contact person (see Table 2).

**Table 2:** Active and passive contact person follow-up and monitoring strategies described in included studies.

Main strategies to follow-up and monitor contact persons	Number of studies reporting type of contact person follow-up and monitoring strategy	Examples
Active strategies		
Regular check-ins via phone calls or home visits	77 (20.4%)	Contact tracers visit or call contact persons or individuals at risk in a systematic cadence, e.g., once or twice per day to carry out symptom checks and, e.g., sputum collection [96–101].
Symptom monitoring	72 (19.0%)	Contact persons are followed up regularly for symptom monitoring and general health and wellbeing checks, either by dedicated staff such as contact tracers, or are asked to self-monitor with regular reporting to contact tracers [102–105].
Systematic testing	55 (14.6%)	Contact persons were tested at least once, in many studies several times (e.g., weekly, at least twice over six months, at T0 and T3 [106–108], etc.) or as soon as they became symptomatic [109–111].
Mandatory isolation and control of its adherence	39 (10.3%)	Contact persons are isolated in their homes [59,92,112–114], or at designated facilities [103,113,115–117]. In some studies ‘compliance officers’ or contact tracers conduct random visits to those in isolation to ensure adherence [89].
Movement surveillance	1 (0.3%)	Two studies reported on movement surveillance by tracking GPS, credit card logs and CCTV [89].
Passive strategies		
Testing referral	19 (5.0%)	Contact persons or those at risk of infection would be advised to undergo testing at health facilities, or self-test at home [118–123].
Recommendation for self-isolation	14 (3.7%)	Contact persons were recommended to self-isolate in their homes, this was often recommended to contact persons with low-risk exposure [68,124–126].
Treatment referral	9 (2.4%)	Symptomatic contact persons or those at risk of infection were referred for treatment or prophylaxis [57,127–129].
Contact person notification via SMS, App, or email	5 (1.3%)	Contact persons would be notified of their status of contact person or being at risk of infection and often advised for self-isolation and/ or testing [88,130,131].

Active follow-up was described in 135 (35.7%) studies and included regular check-ins via phone calls or home visits (20.4%), symptom monitoring (19.0%), systematic testing (14.6%), mandatory isolation and control of its adherence (10.3%) and movement surveillance (0.3%). Passive follow-up was described in 33 (8.7%) studies and included testing referral (5.0%), recommendation for self-isolation (3.7%), treatment referral (2.4%) and contact person notification via SMS, application or email (1.3%). Some studies described several of these methods used for follow-up.

Thirteen studies (3.4%), including 11 (2.9%) on tuberculosis, one (0.3%) on leprosy and one (0.3%) on COVID-19, reported on long follow-up periods. Especially in the tuberculosis and leprosy studies, this often included several tests over many months (i.e., at 6, 12 and 24 months), to monitor for any potential reactivation or late onset of the disease [40,41,66,107,132–139].

#### Enabling adherence to contact tracing strategies.

Across all studies, 28 (7.4%) reported on various support mechanisms to encourage adherence to contact tracing and related measures. Main themes of incentive schemes were identified as financial incentives [7,40,63,95,99,140–149], provision of essential services [99,101,125,150–156], education and information [85,92,157], community support [158], reimbursement of costs incurred [136,144,147], and enforcement measures [61].

Among these 28 studies, only one study, Lu *et al*., evaluated the effectiveness of such an adherence mechanism. The randomized control trials compared a ‘high-touch contact tracing model’ (including incentives by providing social support services) with standard contact tracing, finding that the high-touch contact tracing model increased referral rates to social services by 8.4% (95% confidence interval, 0.8–15.9, p-value = 0.05) showing that social services could be effectively combined with contact tracing to reduce health inequity [156].

### Effectiveness of contact tracing strategies

Among included studies, 330 (87.3%) studies examined contact tracing effectiveness across different disease areas. Previous reviews have discussed the effectiveness of contact tracing, noting that it may be effective for certain diseases in particular contexts (see S7 Table). Only one publication by the Harvard Global Health Institute presented key contact tracing performance indicators with target values (see Table 3) [159].

**Table 3:** List of contact tracing key performance indicators for COVID-19 contact tracing.

Key performance indicators for contact tracing	Goals to be on track for containment
Percentage of positive individuals from tracing vs. symptomatic patients	>80%
Percentage of index cases who indicate contact persons	>75%
Percentage of identified contact persons traced	>90%
Trace time	24 hours
Percentage of contact persons with symptoms at time of trace	close to zero
Percentage of traced contact persons in quarantine, isolation, or active monitoring	90%
Percentage of traced contact persons receiving support	varies with context; locales should set targets
Percentage of traced contact persons assigned to quarantine, isolation, or active monitoring who are fully compliant with program	90%
Percentage of traced contact persons tested	90%
Time from contact tracing program to test of contact person	24 hours

Source: Harvard Global Health Institute (2020) [159].

#### Mapping effectiveness measures across studies.

In the absence of other studies presenting key performance indicators with target values, effectiveness measures were mapped into performance indicators and were categorised into process, output, and impact measures following the conceptual framework of Vogt *et al.* (see Table 4) [68]. Most studies reported the number of contact persons identified (n = 130; 34.4%) and the contact persons screened or tested (n = 111; 29.4%) [68].

**Table 4:** Contact tracing performance indicators across diseases.

Measure of effect and thematic area	Studies that report measure overall (n)
Process	
Number of known contact persons among cases	16
Contact person identification	130
Contact persons screened or tested	111
Timeliness of contact tracing	48
Contact persons initiating prophylactic or curative treatment	45
Contact persons interviewed	21
Contact persons monitored	16
Contact persons quarantined	17
Contact persons vaccinated	8
Output	
Isolation of new cases	2
Reinfection of cases	5
Proportion of contact persons that became cases	187
Contact persons symptomatic	38
Impact	
Incidence reduction	1
Cases prevented	2
Deaths prevented	4

Several studies reported measures of effectiveness that related to specific diseases (i.e., quarantining for COVID-19). The following section describes the indicators that are common across multiple diseases.

#### Timeliness.

Timeliness of contact tracing was assessed in 48 (12.7%) studies. Most of these studies (n = 27; 7.1%) considered time to follow-up and tracing completion. The most common outcome measures included time to contact identification, time between symptom onset and start of isolation or to case investigation.

#### Contact person identification.

Contact person identification was assessed in 130 (34.4%) studies. The number of contact persons identified varied based on the method of contact tracing, individuals executing the tracing, as well as the types of cases and contact persons. Regarding the method of contact tracing, Hong *et al.* demonstrated that the contact tracing team identified more potentially exposed contact persons compared to electronic health record report (median 17 vs 9, p < .001) [160].

As for the types of contact tracers, Mulder *et al.* highlighted that public health nurses in the Netherlands tended to identify more contact persons than recommended in Dutch guidelines for tuberculosis contact person [161]. Similarly, specially trained midwives identified more contact persons for sexually transmissible infections (STI) in Sweden compared to contact tracers that were not specially trained [162]. Likewise, the use of nurse-led contact tracing for hepatitis B virus improved case referral rates by 14% in England [52].

#### Number of known contact persons among cases.

Only 16 (4.2%) studies considered the number of known contact persons among cases, which varied across studies and contexts. For example, one severe acute respiratory syndrome (SARS) contact tracing study from Canada showed that 98.7% of new cases were contact persons of the index case [163]. However, for Ebola, the proportion cases that were registered as contact persons ranged from as low as 6% in Sierra Leone [164] to 31.1% in Guinea. [165] Similarly, Richardus *et al.* demonstrated varying proportions of leprosy cases that were known contact persons, ranging from 90% in Bangladesh to 62% in Thailand [166].

Laxminarayan *et al.* reported a low number of infected contact persons traced to each index case (0.51, 95% confidence interval: 0.49-0.52) for COVID-19 [167]. Across studies, the reported proportion of cases that were known contact persons for COVID-19 ranged from 27.8% from contact tracing in the United States to 80% from contact tracing in Australia [168].

#### Contact persons interviewed.

Overall, 21 (5.6%) studies reported the proportion of contact persons successfully interviewed for contact tracing. Contact persons successfully interviewed ranged from 91.3% for contact tracing of high-risk COVID-19 contact persons in Belgium [169] to only 31% for COVID-19 contact tracing of the general population in the United States [112]. Reasons cited by Kanu *et al.* for not interviewing contact persons included: the contact person did not respond to call attempts (n = 265; 14%), had no available phone number (n = 208; 11%), refused (n = 88; 5%), was a non-resident (n = 20; 1%), or other reasons (n = 406, 22%) [112].

#### Cases prevented.

Two studies (0.5%) considered cases prevented via contact tracing. Duarte *et al.* found that when compared to targeting only close contact persons identified by case interviews, identifying contact persons from home and workplace visits prevented more tuberculosis cases (5 vs 10, respectively) in Portugal.[53] Vaughn *et al.* assessed contact tracing within a university within the United States and estimated that it prevented an additional 132 exposures and 22 COVID-19 infections [170].

#### Deaths prevented.

Four studies (1.1%) considered the association between deaths prevented and contact tracing strategies, all for diseases of airborne or direct transmission. Yalaman *et al.* analysed 138 countries and found that the implementation of comprehensive national contact tracing policies (i.e., contact tracing for each identified case) was significantly associated with a reduction in COVID-19 case fatality (β = −0.13, p < 0.05) [171]. Similarly, in Colombia, Fernández-Niño *et al.* found that tracing of at least five contact persons per COVID-19 case reduces fatality by 48% (95% confidence interval [45,51]). [172] Kanu *et al.* assessed the impact of contact tracing combined with state-mandated stay-at-home orders, public mask mandates, and case investigation in the United States. They determined that this strategy led to an 100% reduction in COVID-19 mortality [112].

Martinson *et al.* examined the risk of death among contact persons that developed tuberculosis comparing home tracing and intensive HIV/ tuberculosis screening or standard of care (clinic referral letters) in South Africa. The deaths in both groups were comparable (1.0% vs 1.2%) [41].

#### Incidence reduction.

Only one (0.3%) study assessed incidence reduction. Kanu *et al*. found that contact tracing led to an 82% reduction in COVID-19 incidence when combined with state-mandated stay-at-home orders, public mask mandates, and case investigation [112].

#### Effectiveness by contact tracing modality.

Seventeen (4.5%) studies assessed the comparative effectiveness of different modalities with varied results (see S8 Table). Memory retrieval techniques during interviews and specialised contact tracers helped identify more contact persons compared to not using these modalities [46,52,162]. Different testing mechanisms influenced the outcomes, such as combining serological rapid diagnostic testing with reverse transcriptase polymerase chain reaction (RT-PCR) leading to a reduction in the number of contact persons needing to quarantine [76]. Involving community health workers increased rates of contact tracing completion [64].

### Socio-cultural factors affecting the effectiveness of contact tracing

Among included studies, 166 (43.9%) studies examined socio-cultural factors that affect contact tracing. Challenges in contact person follow-up have been reported as resistance from the public to quarantining [173]. Certain groups, such as healthcare workers, children, and individuals with high transmission potential, received special attention in some studies, indicating a targeted approach to contact tracing and management [73,103,133,173–178].

Across all studies, 26 (6.9%) reported on various support mechanisms to encourage adherence to contact tracing and related measures. Main themes included financial incentives, provision of essential services, education and information, enforcement measures, community support, and reimbursement for costs. Finally, 14 (3.7%) studies reported that privacy and data security emerged as significant concerns, with attention to protecting patient confidentiality and maintaining safeguards [104,125,179,180]. The perceived intrusiveness of programs, such as active surveillance and movement restrictions, raised concerns about autonomy in certain contexts [133,181,182].

### Health systems governance of contact tracing strategies

#### Implementation.

The review identified 278 (73.5%) studies that examined health systems governance of contact tracing. Contact tracing often involves multiple actors across different levels including international bodies, national governments, provincial authorities, and local health professionals or volunteers (see S5 Fig).

Across studies, 207 (54.8%) reported the level of implementation. Strategies were implemented locally in 97 (25.7%) studies, nationally in 69 (18.3%) studies, and regionally in 41 (10.9%) studies. Strategies described in five (1.3%) studies spanned across countries, typically for air-travel (n = 3), reflecting coordinated efforts and a global approach [80,153,183–185].

Strategies often aligned with wider health policies or responded to specific health crises (e.g., COVID-19, Ebola, SARS). For example, Amisi *et al.* described child tuberculosis contact management within the context of Kenya’s efforts to manage their high rate of tuberculosis [186].

In 17 (4.5%) studies, contact tracing efforts influenced public health interventions. For instance, the findings of a local and domestic Ebola contact tracing strategy prompted the US Centers for Disease Control and Prevention to revise its guidelines on movement and Ebola monitoring [101]. Similarly, a regional tuberculosis strategy resulted in the National Health Security Office of Thailand agreeing to fund tuberculosis screening for household contact persons, irrespective of symptoms [141].

#### Human resources.

In total, 186 (49.2%) studies described the human resources of contact tracing strategies. Strategies often involved multidisciplinary teams, including healthcare staff (physicians, nurses, health workers), public health officials, epidemiologists, field investigators, disease intervention specialists, and community volunteers [99,118,122,125,141,152,153,176,180,187–191].

Several studies emphasised targeted training and ongoing capacity building for effective contact tracing [122,125,152,153,176,187]. Some studies involved such programs to prepare healthcare workers, community health volunteers, and deploy field staff with the necessary skills to effectively carry out contact tracing procedures [99,118,141,153,188]. Four studies noted that the lack of coordination between local management and volunteers, as well as the non-response rate created a substantial workload for volunteers and staff, needing substantial human resources [180,189–191].

Moreover, some studies noted that the resource-intensive nature of contact tracing demanded significant workforce involvement. They required substantial involvement and management from clinical and administrative staff to ensure the quality of the workforce [190]. Studies demonstrated that contact tracing can be emotionally demanding for contact tracers, especially during public health crises [64,133,192,193]. In one study, healthcare workers valued the extra support that stakeholders provided in the implementation of contact tracing to reduce the time and burden on staff [133]. One study from the USA specified a benchmark of 30 tracers per 100,000 population for COVID-19 case investigation and contact tracing [194].

Collaboration across entities, including public health departments and other government agencies, private software developers, academic institutions, and community-based organisations, and non-governmental organisations was a recurring theme.^12,18–22^ Studies suggested that well-trained, cross-functional teams could be efficient in conducting interviews, administering tests, and managing contact tracing processes. Additionally, certain roles, like epidemiologists, data managers, and administrative staff, require specialised skills to ensure effective contact tracing and data management [111,195–197]. Common challenges discussed among studies included staff shortages, workload issues, limited resources (such as delays in testing samples and equipment), and the need for additional support from non-governmental organisations or private practitioners [122,198–200].

Finally, 22 (5.8%) studies noted the use of community health workers and leaders. These studies found that community members had a good understanding of the local context and would be able to provide local insights, including cultural nuances, social dynamics, and community structures [64,70,77,80,96,200]. Studies reported that community engagement built trust, facilitated information exchange, and promoted a more targeted and culturally sensitive approach to contact tracing [59,152]. Cultural competence and language skills were also mentioned to establish rapport with individuals and improve the likelihood of cooperation within communities, especially in studies that focused more on community-based contact tracing [98,194,199]. Research suggested that contact tracers should convey information clearly and gather information from cases on contact persons as well as provide guidance in a supportive manner [118,199,201].

#### Technical resources.

In total, 94 (24.9%) studies included digital methods of contact tracing. Bluetooth and Global Positioning System (GPS) (in COVID-19, Ebola and Mpox studies) technologies were used for proximity tracking [146,202], sometimes this was linked with decentralised data storage for privacy.^8^ Geolocation software utilised GPS (in COVID-19, Ebola, tuberculosis and Pertussis studies) and Closed-Circuit Television (CCTV) data (in COVID-19, Middle East respiratory syndrome (MERS) and Mpox studies) [55,203,204]. Mobile applications (in COVID-19, tuberculosis, Ebola, Chlamydia studies) and exposure notification networks (in COVID-19, tuberculosis and Chlamydia studies) were useful for linking contact persons with cases through exchanging data among users [205]. Cloud-based systems ensured secure data transfer in the majority of studies describing digital methods supporting contact tracing (in COVID-19, Mpox, tuberculosis, Hepatitis B, Ebola studies) [145,152,206]. Data processing algorithms, such as Cross-Industry Standard Process (CRISP) were also used to enhance data collection and analysis (in COVID-19, tuberculosis and Mpox studies)[196]. Sometimes, credit card transaction logs (in COVID-19 and Mpox studies) were used [89]. Additionally, one Syphilis study reported using Facebook to identify contact persons [207].

Elements of manual methods, involving human contact tracers, contact person cards, and home visit workbooks were reported in most included studies (253, 66.9%) studies [166,208–210]. For example some strategies provided invitation cards to index cases to invite their contact persons to participate [54,141,211].

Health information systems like the Calendaring and Scheduling Consortium (CalCONNECT) [156] and District Health Information Software 2 (DHIS2) [212] integrated data platforms were also utilised, involving national health management data platforms for efficient data entry and analysis. Notification systems consisted of automated messaging, and emergency management software for effective communication (in COVID-19 studies) [92,190].

#### Financial resources.

Contact tracing has many financial implications including renumeration, testing and diagnostics, and incentives. Compensation for individuals involved in the contact tracing process includes personnel wages and travel costs associated with commuting to and from their place of employment as well as the cost involved in physically moving around to trace contact [101,141,144]. One (0.3%) study mentioned that delays in payments for tracers decreased their motivation to work [96]. A cost associated with testing and diagnostic equipment such as X-ray machines, as well as laboratory technicians and other individuals who may be involved with testing and diagnostics was reported [63,72]. In some contact tracing strategies, index patients were offered a financial incentive for every contact person they identified, and contact persons were financially compensated if they presented themselves for testing [142,143].

Funding mechanisms were varied and included government funding [213], specific government programs [156,194], non-governmental organisations [133,200], private sector contributions [200], religious institutions [214], individual donors [214], and public health organisations such as the WHO [189].

The cost per case detected was only reported in four (1.1%) studies. Costs ranged from $33 for COVID-19 to $2,200 for HIV. The considerable variability in the data may be attributable to significant differences among the four studies regarding disease focus, the range of costs incorporated in the estimations, country settings, and the time periods analysed.

## Discussion

To our knowledge, this is the first global systematic review to look at contact tracing across all infectious diseases with human-to-human transmission. The review included 378 studies, and examined the definitions, effectiveness, socio-cultural aspects, and health systems governance of contact tracing strategies. Most studies originated from Europe and North America and focused on diseases such as COVID-19 and tuberculosis.

The effectiveness of contact tracing is influenced by several key factors. Effective health systems governance and adherence to established standards and norms are essential for the successful implementation of contact tracing strategies. Adherence by the affected population is critical, with trust playing a significant role [7,215–217]. In line with previous research, this review also highlights that reinforcing community connections through engaging local non-governmental organisations and community leaders can strengthen contact tracing efforts [218]. Additionally, integrating contact tracing with broader public health and social measures may enhance adherence [101,141].

These factors are influenced by the context and disease characteristics, with variability in success rates across different diseases and settings. Disease-specific indicators, such as reinfection rates for STIs, vaccination for hepatitis B, and quarantine measures for COVID-19, were commonly reported. The data identified could be used in the development of contact tracing strategies or guidelines for novel or emerging infectious diseases.

The paucity of evidence regarding output, outcome, and impact indicators for contact tracing effectiveness is a notable finding. This gap constrains our understanding of contact tracing’s efficacy in different epidemic contexts. Addressing this issue is crucial for optimising contact tracing strategies and improving epidemic preparedness. To address this gap, we recommend that researchers and program managers implementing contact tracing interventions should prepare monitoring and evaluation plans that define and enable measurement of input, process, output, outcome, and impact indicators where feasible, as proposed in a scoping review of contact tracing for COVID-19 [219]. In particular, output (e.g., case isolation, contact quarantine) and outcome (transmission reduction) indicators should be prioritised in addition to process indicators that are currently more commonly measured, to enable effectiveness evaluations for contact tracing for different infectious diseases.

In addition to effectiveness, acceptability of contact tracing strategies varied significantly by country and infection type, influenced by factors like stigma and privacy concerns [10–13,100]. These structures were central for enhancing operational efficiency and ensuring the resilience of the contact tracing workforce. However, the review of the literature has shown that the effectiveness of the process is determined by several factors, including the disease characteristics, as well as the social, cultural, economic, and political context where the pathogen is identified and may spread.

Equitable access and community engagement are pivotal, necessitating proactive measures to address disparities and ensure inclusive participation [60,152]. The evolving landscape of digital tools holds transformative potential for contact tracing but requires continuous technological advancements and strict privacy adherence. Although, the effectiveness of digital strategies was beyond the scope of this review.

Our review indicated that contact tracing demands thorough workforce management and significant resources. A recent study by Islam *et al.* highlights similar issues, emphasising the need for contextualised guidance, effective implementation strategies, and comprehensive training to enhance the effectiveness of contact tracing efforts [220]. The study underlines the importance of tailored guidance and ongoing education to address local, social, cultural, and infrastructural nuances and improve the overall impact of contact tracing initiatives, in line with current research and the evidence identified through our review [122,125,152,153,176,187,220,221].

Similarly, collaboration emerged as a key element in contact tracing strategies identified through the review. However, challenges like staff shortages and resource constraints persist. Effective training and robust mental health support for contact tracers are fundamental for sustaining workforce resilience and have been demonstrated as improving contact tracing effectiveness across various contexts [99,118,122,125,141,152,153,176,180,187–191]. Sustained financial investment is required to support the resource-intensive nature of contact tracing. Efficient allocation of resources and prioritisation of contact tracing efforts based on cost-benefit analyses can further enhance effectiveness [133,156,189,194,200,213,214].

### Limitations

This review highlights several limitations. Within the literature base, the predominance of studies from Europe and North America limits generalisability. Moreover, most studies focused on COVID-19 (n = 179; 47.4%) followed by tuberculosis (n = 101; 26.7%). All other diseases were the focus of only 2–12 studies each. The urgency and global impact of the COVID-19 pandemic might have influenced the design, execution, and outcomes of contact tracing strategies, which could limit the applicability of our findings to other epidemics and pathogens with different modes of transmission and epidemiological characteristics. The reliance on descriptive or non-randomised studies underscores the need for more rigorous and comparable operational research methodologies. The number of actual trials included in this review is relatively small compared to the total number of studies. This could potentially limit the robustness of our findings and conclusions, as trials often provide more definitive evidence compared to other types of studies. Additionally, the review does not comprehensively address how factors like outbreak dynamics and disease characteristics impact contact tracing effectiveness in curbing the attack rate and cost-effectiveness of the intervention. Furthermore, the effectiveness of digital contact tracing tools was not explored in depth. Lastly, in the identified evidence for effectiveness of contact tracing strategies, no analysis was conducted to assess the quality of the identified contact persons, including assessments of whether any of them were infected.

### Future research needs

Future research should aim to establish standardised metrics for comparative analysis, explore the impact of contact tracing strategies on disease incidence and mortality, and expand research beyond Europe and North America, and beyond diseases like COVID-19 and tuberculosis. There is a need to optimise community-based approaches to account for local nuances and cultural sensitivities. Evaluating the interplay between health systems governance models, resource allocation, workforce support, and overall effectiveness is essential. Additionally, future research should investigate the long-term effects of integrating contact tracing with broader health policies.

By addressing these gaps, future research can enhance the understanding and implementation of effective contact tracing strategies for novel and emerging infectious diseases.

## Supporting information

S1 Table Search terms for EMBASE and MEDLINE via Embase.com (Elsevier; https://www.embase.com). (DOCX)

S2 Table Search terms for MEDLINE and MEDLINE IN-PROCESS via PubMed (NIH National Library of Medicine, https://pubmed.ncbi.nlm.nih.gov/). (XLSX)

S3 Table Search terms for the Cochrane Methodology Register and Cochrane Database of Systematic Reviews (via the Cochrane Library, https://www.cochranelibrary.com/advanced-search/search-manager). (DOCX)

S4 Table PICO criteria. (DOCX)

S5 Table Risk of bias in included qualitative studies. (DOCX)

S6 Table GRADE CerQual Evidence Profile for qualitative studies. (DOCX)

S7 Table synthesis of previous reviews
(DOCX)

S8 Table Comparative effectiveness of contact tracing strategies. (DOCX)

S1 Fig Disease areas across included studies. Note: Some studies considered more than one disease. “Other single disease” includes methicillin-resistant Staphylococcus aureus (MRSA) infection, mumps, pertussis, severe acute respiratory syndrome (SARS), scarlet fever, (n = 1 for each).
(TIF)

S2 Fig Types of included studies. (TIF)

S3 Fig Risk of bias in included randomized control trials. (PNG)

S4 Fig Risk of bias in included non-randomized studies. (PNG)

S5 Fig Example of governance strategy for COVID-19. (TIF)

S1 Box Contact person definition. (DOCX)

S1 Data WHO contact tracing list of studies: includes all 9935 articles that were found through the search strategy. (XLSX)

S2 Data Data Extraction Template_final_v3.0: includes data from the 378 studies that were retained for the review. (XLSX)
