# Supplementary material for: Contact tracing strategies for infectious diseases: A systematic literature review
Source: PLOS Glob Public Health. 2025 May 9;5(5):e0004579. doi: 10.1371/journal.pgph.0004579 (PMC12063836; doi:10.1371/journal.pgph.0004579)
Supplement: S1 Table — (DOCX) [file pgph.0004579.s001.docx]

S1 Table. Search terms for **EMBASE** and **MEDLINE** via Embase.com (Elsevier; https://[www.embase.com](http://www.embase.com/))

| **No.** | **Query** | **Results** |
| --- | --- | --- |
| **#1*** | 'contact examination'/exp OR 'contact tracing':ti,ab OR 'contact trace':ti,ab OR 'contact tracer':ti,ab OR 'contact detection':ti,ab OR 'contact investigation':ti,ab OR 'contact investigations':ti,ab OR 'contact investigator':ti,ab OR 'contact examination':ti,ab OR 'contact examiner':ti,ab OR 'contact screen':ti,ab OR 'contact screening':ti,ab OR 'contact screener':ti,ab OR 'partner notification':ti,ab OR 'partner notifier':ti,ab OR 'partner notice':ti,ab OR 'contact tracing':ti,ab OR 'contact-tracing':ti,ab OR 'tracing contact*':ti,ab OR 'contact follow-up':ti,ab OR 'case detection*':ti,ab OR 'epidemic investigation*':ti,ab | 6,849 |
| **#2**** | 'disease transmission'/exp OR 'infection'/exp OR 'vector borne disease'/exp OR 'vaccine preventable disease'/exp OR 'disease transmission':ti,ab OR 'disease transmission, infectious':ti,ab OR 'infection transfer':ti,ab OR 'infection transmission':ti,ab OR 'infectious disease transmission':ti,ab OR 'infectious transmission':ti,ab OR 'transmission of infection':ti,ab OR 'transmission of infectious disease':ti,ab OR 'disease transmit*':ti,ab OR 'disease transmissibl*':ti,ab OR 'disease transfer*':ti,ab OR ‘infectious disease’:ti,ab | 4,618,612 |
| **#3** | #1 AND #2 | 14,406 |
| **#4** | #3 AND ('article'/it OR 'article in press'/it) | 9,440 |
| **#5** | #3 AND ‘review’/it | 1,284 |
| **#6** | #4 OR #5 | 10,724 |

*The following terms were adapted from: Hossain, A. D., Jarolimova, J., *et al.* (2022). *The Lancet Public Health*.[1]

**The following terms were adapted from: Kotlyar, A. M., Grechukhina, O., *et al.* (2021). *American Journal of Obstetrics and Gynecology*, 224(1), 35-53.[2] and Lee, M. H., Lee, G. A., *et al.* (2020). *PloS ONE*, 15(3), e0229911.[3]

Date of the search: 8th September 2023

1. Hossain AD, Jarolimova J, Elnaiem A, Huang CX, Richterman A, Ivers LC. Effectiveness of contact tracing in the control of infectious diseases: a systematic review. Lancet Public Health. 2022;7: e259–e273. doi:10.1016/S2468-2667(22)00001-9

2. Kotlyar AM, Grechukhina O, Chen A, Popkhadze S, Grimshaw A, Tal O, et al. Vertical transmission of coronavirus disease 2019: a systematic review and meta-analysis. Am J Obstet Gynecol. 2021;224: 35-53.e3. doi:10.1016/j.ajog.2020.07.049

3. Lee MH, Lee GA, Lee SH, Park Y-H. A systematic review on the causes of the transmission and control measures of outbreaks in long-term care facilities: back to basics of infection control. PloS one. 2020;15: e0229911.
