# Supplementary material for: Contact tracing strategies for infectious diseases: A systematic literature review
Source: PLOS Glob Public Health. 2025 May 9;5(5):e0004579. doi: 10.1371/journal.pgph.0004579 (PMC12063836; doi:10.1371/journal.pgph.0004579)
Supplement: S2 Table — (XLSX) [file pgph.0004579.s002.docx]

S2 Table. Search terms for MEDLINE and MEDLINE IN-PROCESS via PubMed (NIH National Library of Medicine, https://pubmed.ncbi.nlm.nih.gov/)

| **No.** | **Query** | **Results** |
| --- | --- | --- |
| **#1*** | "contact examination"[MeSH Terms] OR “contact tracing”[Title/Abstract] OR “contact detection”[Title/Abstract] OR "contact trace"[Title/Abstract] OR "contact tracer"[Title/Abstract] OR "contact investigation"[Title/Abstract] OR "contact investigations"[Title/Abstract] OR "contact examination"[Title/Abstract] OR "contact screen"[Title/Abstract] OR "contact screening"[Title/Abstract] OR "partner notification"[Title/Abstract] OR "contact-tracing"[Title/Abstract] OR "contact-tracing"[Title/Abstract] OR "tracing contact*"[Title/Abstract] OR "contact follow-up"[Title/Abstract] OR "case detection*"[Title/Abstract] OR "epidemic investigation*"[Title/Abstract] | 13,486 |
| **#2**** | "disease transmission"[All Fields] OR "disease transmission infectious"[All Fields] OR "infection transfer"[All Fields] OR "infection transmission"[All Fields] OR "infectious disease transmission"[All Fields] OR "infectious transmission"[All Fields] OR "transmission of infection"[All Fields] OR "transmission of infectious disease"[All Fields] OR “infectious disease”[All Fields] | 182,256 |
| **#3** | #1 AND #2 | 1,908 |

*The following terms were adapted from: Hossain, A. D., Jarolimova, J., *et al.* (2022). *The Lancet Public Health*.[1]

**The following terms were adapted from: Kotlyar, A. M., Grechukhina, O., *et al.* (2021). *American Journal of Obstetrics and Gynecology*, 224(1), 35-53.[2] and Lee, M. H., Lee, G. A., *et al.* (2020). *PloS ONE*, 15(3), e0229911.[3]

Date of the search: 8th September 2023

1. Hossain AD, Jarolimova J, Elnaiem A, Huang CX, Richterman A, Ivers LC. Effectiveness of contact tracing in the control of infectious diseases: a systematic review. Lancet Public Health. 2022;7: e259–e273. doi:10.1016/S2468-2667(22)00001-9

2. Kotlyar AM, Grechukhina O, Chen A, Popkhadze S, Grimshaw A, Tal O, et al. Vertical transmission of coronavirus disease 2019: a systematic review and meta-analysis. Am J Obstet Gynecol. 2021;224: 35-53.e3. doi:10.1016/j.ajog.2020.07.049

3. Lee MH, Lee GA, Lee SH, Park Y-H. A systematic review on the causes of the transmission and control measures of outbreaks in long-term care facilities: back to basics of infection control. PloS one. 2020;15: e0229911.
