# Supplementary material for: Contact tracing strategies for infectious diseases: A systematic literature review
Source: PLOS Glob Public Health. 2025 May 9;5(5):e0004579. doi: 10.1371/journal.pgph.0004579 (PMC12063836; doi:10.1371/journal.pgph.0004579)
Supplement: S3 Table — (DOCX) [file pgph.0004579.s003.docx]

S3 Table. Search terms for the Cochrane Methodology Register and Cochrane Database of Systematic Reviews (via the Cochrane Library, https://www.cochranelibrary.com/advanced-search/search-manager)

| **No.** | **Query** | **Results** |
| --- | --- | --- |
| **#1** | MeSH descriptor: [Contact Tracing] explode all trees | 131 |
| **#2*** | "contact trace":ti,ab OR "contact tracer":ti,ab OR "contact investigation":ti,ab OR “contact detection”:ti,ab OR “contact tracing”:ti,ab OR "contact investigations":ti,ab OR "contact investigator":ti,ab OR "contact examination":ti,ab OR "contact examiner":ti,ab OR "contact screen":ti,ab OR "contact screening":ti,ab OR "contact screener":ti,ab OR "partner notification":ti,ab OR "partner notifier":ti,ab OR "partner notice":ti,ab OR "contact tracing":ti,ab OR "contact-tracing":ti,ab OR "tracing contact":ti,ab OR "contact follow-up":ti,ab OR "case detection":ti,ab OR "epidemic investigation":ti,ab | 658 |
| **#3** | #1 OR #2 | 695 |
| **#4** | MeSH descriptor: [Disease Transmission, Infectious] explode all trees | 1,282 |
| **#5** | MeSH descriptor: [Infections] explode all trees | 100,845 |
| **#6**** | "vector borne disease" OR "vaccine preventable disease" OR "disease transmission" OR "disease transmission, infectious" OR "infection transfer" OR "infection transmission" OR "infectious disease transmission" OR "infectious transmission" OR "transmission of infection" OR "transmission of infectious disease" OR “infectious disease” | 6,194 |
| **#7** | #4 OR #5 OR #6 | 103,668 |
| **#8** | #3 AND #7 | 412 |
| **#9** | #3 AND #7 Limit: CMR and CDSR | 13 |

*The following terms were adapted from: Hossain, A. D., Jarolimova, J., *et al.* (2022). *The Lancet Public Health*.[1]

**The following terms were adapted from: Kotlyar, A. M., Grechukhina, O., *et al.* (2021). *American Journal of Obstetrics and Gynecology*, 224(1), 35-53.[2] and Lee, M. H., Lee, G. A., *et al.* (2020). *PloS ONE*, 15(3), e0229911.[3]

Date of the search: 8th September 2023

1. Hossain AD, Jarolimova J, Elnaiem A, Huang CX, Richterman A, Ivers LC. Effectiveness of contact tracing in the control of infectious diseases: a systematic review. Lancet Public Health. 2022;7: e259–e273. doi:10.1016/S2468-2667(22)00001-9

2. Kotlyar AM, Grechukhina O, Chen A, Popkhadze S, Grimshaw A, Tal O, et al. Vertical transmission of coronavirus disease 2019: a systematic review and meta-analysis. Am J Obstet Gynecol. 2021;224: 35-53.e3. doi:10.1016/j.ajog.2020.07.049

3. Lee MH, Lee GA, Lee SH, Park Y-H. A systematic review on the causes of the transmission and control measures of outbreaks in long-term care facilities: back to basics of infection control. PloS one. 2020;15: e0229911.
