# Supplementary material for: Contact tracing strategies for infectious diseases: A systematic literature review
Source: PLOS Glob Public Health. 2025 May 9;5(5):e0004579. doi: 10.1371/journal.pgph.0004579 (PMC12063836; doi:10.1371/journal.pgph.0004579)
Supplement: S4 Table — (DOCX) [file pgph.0004579.s004.docx]

S4 Table. PICO criteria

| **PICO Category** | **What are the contact tracing strategies?** | **How effective are the contact tracing strategies?** | **What influences contact tracing strategies?** | **How are contact tracing strategies governed?** |
| --- | --- | --- | --- | --- |
| **Population** | General populations at risk of infectious disease | | | |
| **Intervention/ comparator** | Contact tracing strategies | | | |
| **Outcomes** | - Type of infection - Contact definitions - Main features of the CT program - Activities carried out during the various steps of the CT process - Testing or other follow-up modalities Incentives to encourage adherence | - Number of known contacts among cases - Contact identification - Contacts screened or tested - Timeliness of contact tracing - Contacts initiating prophylactic or curative treatment - Contacts interviewed - Contacts monitored - Contacts quarantined - Contacts vaccinated - Isolation of new cases - Reinfection of cases - Proportion of contacts that became cases - Contacts symptomatic - Incidence reduction - Cases prevented - Deaths prevented | - Geography - Setting - Phase of infection - Disease burden - Community impacts | - Human and technical resources required, including financial and social support to tracers and affected communities - Governance model - Legal/ethical frameworks that underpin strategy - Data sharing and protection measures - Effects on other policies |

Abbreviations: CT: contact tracing; PICO: Population, Intervention, Comparator, Outcome(s)
