# Supplementary material for: Contact tracing strategies for infectious diseases: A systematic literature review
Source: PLOS Glob Public Health. 2025 May 9;5(5):e0004579. doi: 10.1371/journal.pgph.0004579 (PMC12063836; doi:10.1371/journal.pgph.0004579)
Supplement: S5 Table — (DOCX) [file pgph.0004579.s005.docx]

S5 Table. Risk of bias in included qualitative studies

| **Author year** | **D1** | **D2** | **D3** | **D4** | **D5** | **D6** | **Overall** |
| --- | --- | --- | --- | --- | --- | --- | --- |
| **Asare *et al.* 2023**[1] | Yes | Yes | Yes | No | Yes | Yes | No or very minor concerns |
| **Asiimwe *et al.* 2021**[2] | Yes | Yes | Yes | Yes | Yes | Yes | No or very minor concerns |
| **Aslam *et al.* 2023**[3] | Yes | Yes | Yes | No | Yes | Yes | No or very minor concerns |
| **Ayalon *et al.* 2023**[4] | Yes | Yes | Yes | Yes | Yes | Yes | No or very minor concerns |
| **Cochrane *et al.* 2016**[5] | Yes | Yes | Yes | No | Yes | Yes | No or very minor concerns |
| **Danquah *et al.* 2019**[6] | Yes | Yes | Yes | No | Yes | Yes | No or very minor concerns |
| **DePhilippis *et al.* 1992**[7] | Partially | Partially | Partially | No | Unclear | No | Serious concerns |
| **Dowthwaite *et al.* 2022**[8] | Yes | Partially | Yes | No | Yes | Yes | Minor concerns |
| **Fawole *et al.* 2023**[9] | Yes | Yes | Yes | No | Yes | Yes | No or very minor concerns |
| **Goroh *et al.* 2023**[10] | Yes | Yes | Yes | Yes | Yes | Yes | No or very minor concerns |
| **Greiner *et al.* 2015**[11] | Yes | Yes | Partially | No | Yes | Yes | Minor concerns |
| **Hoang *et al.* 2023**[12] | Yes | Yes | Yes | Yes | Yes | Yes | No or very minor concerns |
| **Hollis *et al.* 2023**[13] | Yes | Yes | Yes | Yes | Yes | Yes | No or very minor concerns |
| **Kas-Osoka *et al.* 2022**[14] | Yes | Partially | Yes | No | Yes | Yes | Minor concerns |
| **Lucivero *et al.* 2022**[15] | Yes | Partially | Yes | Unclear | Yes | Unclear | Moderate concerns |
| **McCarthy *et al.* 2007**[16] | Yes | Yes | Partially | No | Yes | No | Moderate concerns |
| **Mulder *et al.* 2012**[17] | Yes | Yes | Yes | No | Yes | Yes | No or very minor concerns |
| **Samuel *et al.* 2022**[18] | Yes | Partially | Yes | No | Yes | Yes | Minor concerns |
| **Tesfaye *et al.* 2020**[19] | Yes | Yes | Yes | Yes | Yes | Yes | No or very minor concerns |
| **van der Meer *et al.* 2023**[20] | Yes | Yes | Yes | Yes | Yes | Yes | No or very minor concerns |
| **Vo *et al.* 2023**[21] | Yes | Yes | Yes | No | Yes | Yes | No or very minor concerns |
| **Woodward & Rivers 2023**[22] | Yes | Yes | Yes | No | Yes | Yes | No or very minor concerns |

Definition of domains of the risk of bias assessment

**D1**: Setting and context

**D2**: Sampling strategy

**D3**: Data analysis

**D4**: Evidence to support claims

**D5**: Reflexivity

**D6**: Sensitivity to ethical concerns

1. Asare IT, Douglas M, Kye-Duodu G, Manu E. Challenges and opportunities for improved contact tracing in Ghana: experiences from Coronavirus disease-2019-related contact tracing in the Bono region. BMC Infect Dis. 2023;23: 335. doi:10.1186/s12879-023-08317-6

2. Asiimwe N, Tabong PT-N, Iro SA, Noora CL, Opoku-Mensah K, Asampong E. Stakeholders perspective of, and experience with contact tracing for COVID-19 in Ghana: A qualitative study among contact tracers, supervisors, and contacts. PLoS One. 2021;16: e0247038. doi:10.1371/journal.pone.0247038

3. Aslam MA, Murtaza M, Zakar R, Rashid J. Insights from Government Officials on Strategies and Practices for Managing the Covid-19 Pandemic: A Qualitative Study. Pakistan Journal of Medical & Health Sciences. 2023;17: 691–691. doi:10.53350/pjmhs2023171691

4. Ayalon O, Li S, Preneel B, Redmiles EM. Not Only for Contact Tracing: Use of Belgium’s Contact Tracing App among Young Adults. Proc ACM Interact Mob Wearable Ubiquitous Technol. 2023;6: 202:1-202:26. doi:10.1145/3570348

5. Cochrane A, Collins P, Horwood JP. Barriers and opportunities for hepatitis B testing and contact tracing in a UK Somali population: a qualitative study. Eur J Public Health. 2016;26: 389–395. doi:10.1093/eurpub/ckv236

6. Danquah LO, Hasham N, MacFarlane M, Conteh FE, Momoh F, Tedesco AA, et al. Use of a mobile application for Ebola contact tracing and monitoring in northern Sierra Leone: a proof-of-concept study. BMC Infect Dis. 2019;19: 810. doi:10.1186/s12879-019-4354-z

7. DePhilippis D, Metzger DS, Woody GE, Navaline HA. Attitudes toward mandatory human immunodeficiency virus testing and contact tracing. A survey of intravenous drug users in treatment. J Subst Abuse Treat. 1992;9: 39–42. doi:10.1016/0740-5472(92)90008-c

8. Dowthwaite L, Wagner HG, Babbage CM, Fischer JE, Barnard P, Nichele E, et al. The relationship between trust and attitudes towards the COVID-19 digital contact-tracing app in the UK. PLoS One. 2022;17: e0276661. doi:10.1371/journal.pone.0276661

9. Fawole OI, Bello S, Adebowale AS, Bamgboye EA, Salawu MM, Afolabi RF, et al. COVID-19 surveillance in Democratic Republic of Congo, Nigeria, Senegal and Uganda: strengths, weaknesses and key Lessons. BMC Public Health. 2023;23: 835. doi:10.1186/s12889-023-15708-6

10. Goroh MMD, van den Boogaard CHA, Lukman KA, Lowbridge C, Juin WK, William T, et al. Factors affecting implementation of tuberculosis contact investigation and tuberculosis preventive therapy among children in Sabah, East Malaysia: A qualitative study. PLoS One. 2023;18: e0285534. doi:10.1371/journal.pone.0285534

11. Greiner AL, Angelo KM, McCollum AM, Mirkovic K, Arthur R, Angulo FJ. Addressing contact tracing challenges-critical to halting Ebola virus disease transmission. Int J Infect Dis. 2015;41: 53–55. doi:10.1016/j.ijid.2015.10.025

12. Hoang PA, Tran NT, Nguyen THH, Nguyen TTH. Barriers to COVID-19 contact tracing: View from frontline healthcare students in Vietnam. Public Health Nurs. 2023;40: 528–534. doi:10.1111/phn.13189

13. Hollis S, Stolow J, Rosenthal M, Morreale SE, Moses L. Go.Data as a digital tool for case investigation and contact tracing in the context of COVID-19: a mixed-methods study. BMC Public Health. 2023;23: 1717. doi:10.1186/s12889-023-16120-w

14. Kas-Osoka C, Moss J, Alexander L, Davis J, Parham I, Barre I, et al. African Americans views of COVID-19 contact tracing and testing. Am J Infect Control. 2022;50: 577–580. doi:10.1016/j.ajic.2022.02.032

15. Lucivero F, Marelli L, Hangel N, Zimmermann BM, Prainsack B, Galasso I, et al. Normative positions towards COVID-19 contact-tracing apps: findings from a large-scale qualitative study in nine European countries. Critical Public Health. 2022;32: 5. doi:10.1080/09581596.2021.1925634

16. McCarthy M, Haddow LJ, Furner V, Mindel A. Contact tracing for sexually transmitted infections in New South Wales, Australia. Sex Health. 2007;4: 21–25. doi:10.1071/sh06019

17. Mulder C, Harting J, Jansen N, Borgdorff MW, van Leth F. Adherence by Dutch public health nurses to the national guidelines for tuberculosis contact investigation. PLoS One. 2012;7: e49649. doi:10.1371/journal.pone.0049649

18. Samuel G, Roberts SL, Fiske A, Lucivero F, McLennan S, Phillips A, et al. COVID-19 contact tracing apps: UK public perceptions. Crit Public Health. 2022;32: 31–43. doi:10.1080/09581596.2021.1909707

19. Tesfaye L, Lemu YK, Tareke KG, Chaka M, Feyissa GT. Exploration of barriers and facilitators to household contact tracing of index tuberculosis cases in Anlemo district, Hadiya zone, Southern Ethiopia: Qualitative study. PLoS One. 2020;15: e0233358. doi:10.1371/journal.pone.0233358

20. van der Meer A, Helms YB, Baron R, Crutzen R, Timen A, Kretzschmar MEE, et al. Citizen involvement in COVID-19 contact tracing with digital tools: a qualitative study to explore citizens’ perspectives and needs. BMC Public Health. 2023;23: 1804. doi:10.1186/s12889-023-16664-x

21. Vo AV, Majnoonian A, Ni J, Garfein RS, Wishard Guerra A, Fielding-Miller R. Challenges of COVID-19 Case Investigation and Contact Tracing in School Settings: An Initial Investigation. J Sch Health. 2023;93: 353–359. doi:10.1111/josh.13308

22. Woodward A, Rivers C. Case Investigation and Contact Tracing in US State and Local Public Health Agencies: Sustaining Capacities and Applying Lessons Learned From the COVID-19 Pandemic and 2022 Mpox Outbreak. Health Secur. 2023;21: S8–S16. doi:10.1089/hs.2023.0011
