# Supplementary material for: Contact tracing strategies for infectious diseases: A systematic literature review
Source: PLOS Glob Public Health. 2025 May 9;5(5):e0004579. doi: 10.1371/journal.pgph.0004579 (PMC12063836; doi:10.1371/journal.pgph.0004579)
Supplement: S6 Table — (DOCX) [file pgph.0004579.s006.docx]

S6 Table. GRADE CerQual Evidence Profile for qualitative studies

| **Key finding** | **Studies supporting key finding** | **Methodological quality** | **Relevance-research question** | **Relevance-population** | **Coherence** | **Adequacy-reviews** | **Adequacy-primary studies** | **Overall assessment of confidence** |
| --- | --- | --- | --- | --- | --- | --- | --- | --- |
| **People have concerns about data security and privacy impacting trust in contact tracing strategies.** | 10 studies [1–10] | Moderate methodological concerns | Full (10/10) | Full (10/10) | High coherence | NA | Minor adequacy concerns | Moderate confidence |
| **Privacy issues, government surveillance fears, and the need for compatibility with existing privacy regulations are common ethical concerns,** | 16 studies [4,5,9–22] | Moderate methodological concerns | Full (16/16) | Full (16/16) | High coherence | NA | Minor adequacy concerns | Moderate confidence |
| **For COVID-19 privacy, autonomy, and data security were highlighted as the main factors for intrusiveness** | 40 studies | Moderate methodological concerns | Full (40/40) | Full (40/40) | High coherence | NA | Moderate adequacy concerns | Low confidence |
| **For Ebola direct active surveillance and movement restrictions were highlighted as intrusive measures** | 1 study [23] | Moderate methodological concerns | Full (1/1) | Full (1/1) | High coherence | NA | Moderate adequacy concerns | Low confidence |
| **For TB intrusiveness is reflected in challenges such as reluctance to provide correct contact information, the use of consent-based approach to minimize intrusiveness and parental refusal indicating concerns about autonomy** | 4 studies [24–27] | Moderate methodological concerns | Full (4/4) | Full (4/4) | High coherence | NA | Moderate adequacy concerns | Low confidence |
| **Factors influencing acceptability include privacy concerns, surveillance, and mode of implementation.** | 8 studies [1,2,16,28–32] | Moderate methodological concerns | Full (8/8) | Full (8/8) | High coherence | NA | Moderate adequacy concerns | Low confidence |
| **In Ebola there are challenges in monitoring and movement restrictions associated with adherence.** | 2 studies [23,33] | Moderate methodological concerns | Full (2/2) | Full (2/2) | High coherence | NA | Moderate adequacy concerns | Low confidence |

Abbreviations: NA: not applicable; TB: tuberculosis.

1. Garrett PM, White JP, Lewandowsky S, Kashima Y, Perfors A, Little DR, et al. The acceptability and uptake of smartphone tracking for COVID-19 in Australia. PLoS One. 2021;16: e0244827. doi:10.1371/journal.pone.0244827

2. Osmanlliu E, Paquette J, Rodriguez Duarte MA, Bédard S, de Marcellis-Warin N, Zhegu M, et al. Public Perspectives on Exposure Notification Apps: A Patient and Citizen Co-Designed Study. J Pers Med. 2022;12: 729. doi:10.3390/jpm12050729

3. Fields VL, Kiphibane T, Eason JT, Hafoka SF, Lopez AS, Schwartz A, et al. Assessment of contact tracing for COVID-19 among people experiencing homelessness, Salt Lake County Health Department, March-May 2020. Ann Epidemiol. 2021;59: 50–55. doi:10.1016/j.annepidem.2021.04.002

4. Dowthwaite L, Fischer J, Perez Vallejos E, Portillo V, Nichele E, Goulden M, et al. Public Adoption of and Trust in the NHS COVID-19 Contact Tracing App in the United Kingdom: Quantitative Online Survey Study. J Med Internet Res. 2021;23: e29085. doi:10.2196/29085

5. Touzani R, Schultz E, Vandentorren S, Arwidson P, Guillemin F, Bouhnik A-D, et al. Digital contact tracing during the COVID-19 pandemic in France: Associated factors and reasons for non-use. Int J Med Inform. 2023;171: 104994. doi:10.1016/j.ijmedinf.2023.104994

6. Dowthwaite L, Wagner HG, Babbage CM, Fischer JE, Barnard P, Nichele E, et al. The relationship between trust and attitudes towards the COVID-19 digital contact-tracing app in the UK. PLoS One. 2022;17: e0276661. doi:10.1371/journal.pone.0276661

7. Bachtiger P, Adamson A, Quint JK, Peters NS. Belief of having had unconfirmed Covid-19 infection reduces willingness to participate in app-based contact tracing. NPJ Digit Med. 2020;3: 146. doi:10.1038/s41746-020-00357-5

8. Woodward A, Rivers C. Building case investigation and contact tracing programs in U.S. state and local health departments: a conceptual framework. Public and Global Health; 2023 Jan. doi:10.1101/2023.01.07.23284294

9. Zimmermann BM, Fiske A, Prainsack B, Hangel N, McLennan S, Buyx A. Early Perceptions of COVID-19 Contact Tracing Apps in German-Speaking Countries: Comparative Mixed Methods Study. J Med Internet Res. 2021;23: e25525. doi:10.2196/25525

10. Huang Z, Guo H, Lim HY-F, Chow A. Determinants of the acceptance and adoption of a digital contact tracing tool during the COVID-19 pandemic in Singapore. Epidemiol Infect. 2022;150: e54. doi:10.1017/S0950268822000401

11. van der Meer A, Helms YB, Baron R, Crutzen R, Timen A, Kretzschmar MEE, et al. Citizen involvement in COVID-19 contact tracing with digital tools: a qualitative study to explore citizens’ perspectives and needs. BMC Public Health. 2023;23: 1804. doi:10.1186/s12889-023-16664-x

12. Zirbes J, Sterr CM, Steller M, Dapper L, Nonnenmacher-Winter C, Günther F. Development of a web-based contact tracing and point-of-care-testing workflow for SARS-CoV-2 at a German University Hospital. Antimicrob Resist Infect Control. 2021;10: 102. doi:10.1186/s13756-021-00971-2

13. Santos A, Nuñez G, La S, Dorsey JH, Patel SS, Luk KG, et al. Digital Point Solutions for Extending Contact Tracing Capacity. Sex Transm Dis. 2023;50: S41–S47. doi:10.1097/OLQ.0000000000001712

14. Montagni I, Roussel N, Thiébaut R, Tzourio C. Health Care Students’ Knowledge of and Attitudes, Beliefs, and Practices Toward the French COVID-19 App: Cross-sectional Questionnaire Study. J Med Internet Res. 2021;23: e26399. doi:10.2196/26399

15. Safer M, Letaief H, Hechaichi A, Harizi C, Dhaouadi S, Bouabid L, et al. Identification of transmission chains and clusters associated with COVID-19 in Tunisia. BMC Infect Dis. 2021;21: 453. doi:10.1186/s12879-021-06107-6

16. Kas-Osoka C, Moss J, Alexander L, Davis J, Parham I, Barre I, et al. African Americans views of COVID-19 contact tracing and testing. Am J Infect Control. 2022;50: 577–580. doi:10.1016/j.ajic.2022.02.032

17. Vo AV, Majnoonian A, Ni J, Garfein RS, Wishard Guerra A, Fielding-Miller R. Challenges of COVID-19 Case Investigation and Contact Tracing in School Settings: An Initial Investigation. J Sch Health. 2023;93: 353–359. doi:10.1111/josh.13308

18. Samuel G, Roberts SL, Fiske A, Lucivero F, McLennan S, Phillips A, et al. COVID-19 contact tracing apps: UK public perceptions. Crit Public Health. 2022;32: 31–43. doi:10.1080/09581596.2021.1909707

19. Hassan H, Abo ElSood H, Abd ElGawad B, Kamel R, Fahim M, El Shourbagy S, et al. The value of contact tracing and isolation in mitigation of COVID-19 epidemic: findings from outbreak investigation of COVID-19 onboard Nile Cruise Ship, Egypt, March 2020. BMJ Glob Health. 2022;7: e008681. doi:10.1136/bmjgh-2022-008681

20. COVID-19 National Emergency Response Center, Epidemiology & Case Management Team, Korea Centers for Disease Control & Prevention. Contact Transmission of COVID-19 in South Korea: Novel Investigation Techniques for Tracing Contacts. Osong Public Health Res Perspect. 2020;11: 60–63. doi:10.24171/j.phrp.2020.11.1.09

21. Rodríguez P, Graña S, Alvarez-León EE, Battaglini M, Darias FJ, Hernán MA, et al. A population-based controlled experiment assessing the epidemiological impact of digital contact tracing. Nat Commun. 2021;12: 587. doi:10.1038/s41467-020-20817-6

22. Huang Z, Tay E, Wee D, Guo H, Lim HY-F, Chow A. Public Perception of the Use of Digital Contact-Tracing Tools After the COVID-19 Lockdown: Sentiment Analysis and Opinion Mining. JMIR Form Res. 2022;6: e33314. doi:10.2196/33314

23. Chung WM, Smith JC, Weil LM, Hughes SM, Joyner SN, Hall EM, et al. Active Tracing and Monitoring of Contacts Associated With the First Cluster of Ebola in the United States. Ann Intern Med. 2015;163: 164–173. doi:10.7326/M15-0968

24. Hoang TTT, Nguyen VN, Dinh NS, Thwaites G, Nguyen TA, van Doorn HR, et al. Active contact tracing beyond the household in multidrug resistant tuberculosis in Vietnam: a cohort study. BMC Public Health. 2019;19: 241. doi:10.1186/s12889-019-6573-z

25. Mandalakas AM, Ngo K, Alonso Ustero P, Golin R, Anabwani F, Mzileni B, et al. BUTIMBA: Intensifying the Hunt for Child TB in Swaziland through Household Contact Tracing. PLoS One. 2017;12: e0169769. doi:10.1371/journal.pone.0169769

26. Kelly AM, D’Agostino JF, Andrada LV, Liu J, Larson E. Delayed tuberculosis diagnosis and costs of contact investigations for hospital exposure: New York City, 2010-2014. Am J Infect Control. 2017;45: 483–486. doi:10.1016/j.ajic.2016.12.017

27. Ling D-L, Liaw Y-P, Lee C-Y, Lo H-Y, Yang H-L, Chan P-C. Contact investigation for tuberculosis in Taiwan contacts aged under 20 years in 2005. The International Journal of Tuberculosis and Lung Disease. 2011;15: 50–55.

28. Asiimwe N, Tabong PT-N, Iro SA, Noora CL, Opoku-Mensah K, Asampong E. Stakeholders perspective of, and experience with contact tracing for COVID-19 in Ghana: A qualitative study among contact tracers, supervisors, and contacts. PLoS One. 2021;16: e0247038. doi:10.1371/journal.pone.0247038

29. Horvath L, Banducci S, Blamire J, Degnen C, James O, Jones A, et al. Adoption and continued use of mobile contact tracing technology: multilevel explanations from a three-wave panel survey and linked data. BMJ Open. 2022;12: e053327. doi:10.1136/bmjopen-2021-053327

30. Toffolutti V, Plach S, Maksimovic T, Piccitto G, Mascherini M, Mencarini L, et al. The association between COVID-19 policy responses and mental well-being: Evidence from 28 European countries. Soc Sci Med. 2022;301: 114906. doi:10.1016/j.socscimed.2022.114906

31. Limaye N, Ninesling B, Marcelin F, Nolan C, Sobba W, Hing M, et al. COVID-19 Pandemic Response in a Migrant Farmworker Community: Excess Mortality, Testing Access and Contact Tracing in Immokalee, Florida. Ann Glob Health. 2022;88: 77. doi:10.5334/aogh.3859

32. Kozyreva A, Lorenz-Spreen P, Lewandowsky S, Garrett PM, Herzog SM, Pachur T, et al. Psychological factors shaping public responses to COVID-19 digital contact tracing technologies in Germany. Sci Rep. 2021;11: 18716. doi:10.1038/s41598-021-98249-5

33. Greiner AL, Angelo KM, McCollum AM, Mirkovic K, Arthur R, Angulo FJ. Addressing contact tracing challenges-critical to halting Ebola virus disease transmission. Int J Infect Dis. 2015;41: 53–55. doi:10.1016/j.ijid.2015.10.025
