# Supplementary material for: Contact tracing strategies for infectious diseases: A systematic literature review
Source: PLOS Glob Public Health. 2025 May 9;5(5):e0004579. doi: 10.1371/journal.pgph.0004579 (PMC12063836; doi:10.1371/journal.pgph.0004579)
Supplement: S7 Table — synthesis of previous reviews (DOCX) [file pgph.0004579.s007.docx]

S7 Table. synthesis of previous reviews

| **Author year** | **Aims/objectives** | **Key findings** |
| --- | --- | --- |
| **Rafferty *et al.* 2023**[1] | To provide evidence for SARS-CoV-2 controlling. | H1N1 influenza A virus had the highest reported pooled attack rate per 100 persons (AR = 1.17), followed by SARS-CoV-2 (AR = 0.54) and SARS-CoV (AR = 0.32), Mycobacterium tuberculosis (TB, AR = 0.25), and measles virus (AR = 0.09). There was high heterogeneity in estimates between studies, except for TB. |
| **Velen *et al.* 2021**[2] | To evaluate the effectiveness of contact investigation. | Contact investigation was effective in high-burden settings. Higher pooled prevalence estimates of microbiologically confirmed TB compared with previous reviews. |
| **Ayouni *et al.* 2021**[3] | To evaluate the implemented public health interventions to control COVID-19. | Contact tracing and testing were effective in mitigating the spread of COVID-19. |
| **Pozo-Martin *et al.* 2023**[4] | To evaluate whether and what types of contact tracing interventions were effective in COVID-19 pandemic. | Highly effective contact tracing policies: (1) manual contact tracing with high tracing coverage and either medium-term immunity, highly efficacious isolation/quarantine and/ or physical distancing (2) hybrid manual and digital contact tracing with high app adoption with highly effective isolation/ quarantine and social distancing, (3) secondary contact tracing, (4) eliminating contact tracing delays, (5) bidirectional contact tracing, (6) contact tracing with high coverage in reopening educational institutions. |
| **Juneau *et al.* 2023**[5] | To examine contact tracing effectiveness. | To stop the spread of COVID-19, 2–3 days of delay from a new case develops symptoms to isolate the case and quarantine at least 80% of its contacts. |
| **Littlecott *et al.* 2023**[6] | To assess the effectiveness of general population testing, contact tracing, isolation, and quarantine in reducing SARS-CoV-2 transmission. | Contact tracing in addition to other policies was effective that a significant decline in transmission was observed. |
| **Bannister-Tyrrell *et al.* 2023**[7] | To document experiences and lessons learned from the use of CT interventions. | Adoption ranged from 14.6% to 92.7% in different populations and epidemiological contexts. Trust in authorities, and privacy concerns and beliefs, were the most frequent determinants of adoption and use. |
| **Hossain *et al.* 2022**[8] | To evaluate the effectiveness of contact tracing. | Contact tracing could be effective. However, the ability of authorities to make informed choices might be limited. |
| **Mbivnjo *et al.* 2022**[9] | To examine the effectiveness of contact tracing in reducing measles spread during outbreaks in low- and middle-income countries (LMICs). | The cost dominated by CT in terms of labor of health sector and productivity losses for households. However, overall outbreak expenditure can be low if CT results in fewer and less severe measles cases and a short outbreak duration. |
| **Sagili *et al.* 2022**[10] | To summarize latent tuberculosis infection (LTBI) management strategies among household contacts of TB. | Median 99% (IQR 82%–100%) of contacts were screened. Nine broad themes related to diagnostic testing, health system structure and functions, risk perception, documentation and adherence were considered likely to influence the LTBI care. |
| **Glennie *et al.* 2021**[11] | We therefore conducted a systematic review of active case detection (ACD) methods for leprosy, a condition with similar ACD requirements, to consider how findings could be informative to crusted scabies detection. | Contact tracing and community screening of marginalized ethnic groups yielded the highest new case detection rates. |
| **Jenniskens *et al.* 2021**[12] | To systematically review evidence on effectiveness of contact tracing apps for SARS-CoV-2 on epidemiological and clinical outcomes. | Most studies demonstrated beneficial effects of contact tracing apps on R, total number of infections and mortality rate. |
| **Thomas Craig *et al.* 2021**[13] | To identify and synthesize evidence of effectiveness of contact tracing on infectious viral disease. | Contact tracing was mostly evaluated in combination with other nonpharmaceutical interventions and/or pharmaceutical interventions. Results of studies were highly dependent on epidemic severity (R0 value), number of contacts traced, timeliness, duration, and compliance with combined interventions. Contact tracing effectiveness was particularly limited by logistical challenges associated with increased outbreak size and speed of infection spread. Timely deployment of contact tracing strategically layered with other nonpharmaceutical interventions could be an effective policy. |
| **Chung *et al.* 2021**[14] | To systematically learn lessons from the experiences of countries implementing find, test, trace, isolate, support (FTTIS) in the first wave of the COVID-19 pandemic. | Digital tools such as apps, GPS and mobile geopositioning data have been deployed effectively by some countries for contact tracing and to monitor compliance with self-isolation. |
| **Mazza *et al.* 2021**[15] | To understand the global employment of contact tracing tools and its impact on the prevention and control of the COVID-19 pandemic. | CT apps download rates ranged from 0.01% to 58.3%. Limited data is available on its effectiveness against COVID-19. |
| **Velleca *et al.* 2021**[16] | To update and enhance the estimates of the yield of TB contact investigation in LMIC. | Secondary cases of all active TB 2.87%, bacteriologically confirmed active TB 2.04% and LTBI 43.83%. While the data are heterogenous, these findings can inform strategic and programmatic planning for scale up of TB contact investigation activities. |
| **Anglemyer *et al.* 2020**[17] | To assess the benefits, harms, and acceptability of personal digital contact tracing solutions for identifying contacts of an identified positive case of an infectious disease. | The effectiveness of digital solutions is largely unproven as there are very few published data in real‐world outbreak settings. Modelling studies provide low‐certainty evidence of a reduction in secondary cases if digital contact tracing is used together with other public health measures such as self‐isolation. Cohort studies provide very low‐certainty evidence that digital contact tracing may produce more reliable counts of contacts and reduce time to complete contact tracing. Digital solutions may have equity implications for at‐risk populations with poor internet access and poor access to digital technology. |
| **Taleghani *et al.* 2019**[18] | To review the recent literature evaluating the acceptability and efficacy of partner notification strategies (i.e. direct patient referral, provider referral, or expedited partner treatment) for curable STIs in sub-Saharan Africa. | Proportion of index cases (n = 4163) who successfully notified sex partner(s) was 53% (range 23-95%). Among those who notified (n = 1727), 25% (range 0–77%) had partner(s) that sought evaluation (95% CI 0.51–0.54; 95% CI 0.23–0.27). |
| **Field *et al.* 2023**[19] | To describe ways in which linked data has been used to assist in the response for acute infectious disease events (i.e., outbreaks/epidemics or pandemics). | For outbreak response, data linkage was shown to improve case finding in multiple studies. |
| **Carter *et al.* 2021**[20] | To determine which factors influence symptom reporting during an emerging infectious disease outbreak. | Seven themes were identified as barriers to symptom reporting or accessing healthcare: lack of knowledge of the disease and its treatment, fear of the disease and fear of subsequent treatments or requirements, stigmatization attached to having a disease, invasion of privacy, low concern about symptoms, economic consequences of disease diagnosis, and challenges related to attending a healthcare facility. |
| **Mathevet *et al.* 2021**[21] | To determine if and how health inequities were included in the design of contact tracing interventions in epidemic settings. | The use of tools/concepts for incorporating health inequities, such as the REFLEX-ISS tool,  and ‘proportionate universalism’ when designing contact tracing strategies. |
| **Megnin-Viggars *et al.* 2020**[22] | To identify facilitators and barriers to uptake of, and engagement with, contact tracing during infectious disease outbreaks. | Four themes were identified as facilitators of contact tracing: collective responsibility; personal benefit; co-production of contact tracing systems; and the perception of the system as efficient, rigorous, and reliable. Five themes were identified as barriers to the uptake of, and engagement with, contact tracing: privacy concerns; mistrust and/or apprehension; unmet need for more information and support; fear of stigmatization; and mode-specific challenges. |
| **Amicosante *et al.* 2023**[23] | To provide a comprehensive picture of the organizational aspects of CT activities during the first wave of the pandemic through the systematic identification and description of CT strategies used in different settings. | Outreach activities were shown to increase effectiveness of contact tracing in susceptible groups. |
| **Oyibo *et al.* 2022**[24] | To uncover the key factors that facilitate or militate against the adoption of CTAs, which researchers, designers, and other stakeholders should focus on in future iterations to increase their adoption and effectiveness in curbing the spread of COVID-19. | The identified factors were thematically grouped into ten categories: privacy and trust, app utility, facilitating conditions, social-cognitive factors, ethical concerns, perceived technology threats, perceived health threats, technology familiarity, persuasive design, and sociodemographic factors. Privacy concern was the most frequent factor of CTA adoption, followed by perceived benefit, perceived trust, and perceived data security risk. |
| **Skarp *et al.* 2021**[25] | To evaluate the outbreak costings and simulation studies related to several non-pharmaceutical intervention (NPI) strategies, including isolating, contact tracing and quarantine, and school closures. | Cost related to contact tracing and quarantine: US$40.73 to US$93.59 per contact. |
| **Pegollo *et al.* 2021**[26] | To investigate the acceptance of contact tracing apps. | Assessing DCT acceptance in different settings, populations, and cultural contexts it is of fundamental importance in strategy design. |
| **Akinbi *et al.* 2021**[27] | To review the current challenges of contact tracing apps for COVID-19 in neo-liberal societies, discuss recommendations to address these challenges and explore future directions of the tools. | Privacy concern is the most significant barrier of contact tracing apps in neo-liberal societies, which is largely influenced by the cultural differences, lack of evidence of benefits and growing concerns that government agencies misuse the data. |

Abbreviations: ACD: active case detection; AR: Attack Rate; CI: confidence interval; CT: contact tracing; CTA: contact tracing apps; DCT: digital contact tracing; FTTIS: find, test, trace, isolate, support; GPS: Global Positioning System; IQR: interquartile range; LMIC: low- and middle-income country; LTBI: latent tuberculosis infection; NPI: non-pharmaceutical intervention; SARS-CoV-2: severe acute respiratory syndrome coronavirus 2; STI: sexually transmitted infection; TB: tuberculosis; US: United States.

1. Rafferty AC, Bofkin K, Hughes W, Souter S, Hosegood I, Hall RN, et al. Does 2x2 airplane passenger contact tracing for infectious respiratory pathogens work? A systematic review of the evidence. PLoS One. 2023;18: e0264294. doi:10.1371/journal.pone.0264294

2. Velen K, Shingde RV, Ho J, Fox GJ. The effectiveness of contact investigation among contacts of tuberculosis patients: a systematic review and meta-analysis. Eur Respir J. 2021;58: 2100266. doi:10.1183/13993003.00266-2021

3. Ayouni I, Maatoug J, Dhouib W, Zammit N, Fredj SB, Ghammam R, et al. Effective public health measures to mitigate the spread of COVID-19: a systematic review. BMC Public Health. 2021;21: 1015. doi:10.1186/s12889-021-11111-1

4. Pozo-Martin F, Beltran Sanchez MA, Müller SA, Diaconu V, Weil K, El Bcheraoui C. Comparative effectiveness of contact tracing interventions in the context of the COVID-19 pandemic: a systematic review. Eur J Epidemiol. 2023; 243–266.

5. Juneau C-E, Briand A-S, Collazzo P, Siebert U, Pueyo T. Effective contact tracing for COVID-19: A systematic review. Global Epidemiology. 2023; 100103.

6. Littlecott H, Herd C, O’Rourke J, Chaparro LT, Keeling M, James Rubin G, et al. Effectiveness of testing, contact tracing and isolation interventions among the general population on reducing transmission of SARS-CoV-2: a systematic review. Philosophical Transactions of the Royal Society A: Mathematical, Physical and Engineering Sciences. 2023;381: 20230131. doi:10.1098/rsta.2023.0131

7. Bannister-Tyrrell M, Chen M, Choi V, Miglietta A, Galea G. Systematic scoping review of the implementation, adoption, use, and effectiveness of digital contact tracing interventions for COVID-19 in the Western Pacific Region. Lancet Reg Health West Pac. 2023;34: 100647. doi:10.1016/j.lanwpc.2022.100647

8. Hossain AD, Jarolimova J, Elnaiem A, Huang CX, Richterman A, Ivers LC. Effectiveness of contact tracing in the control of infectious diseases: a systematic review. Lancet Public Health. 2022;7: e259–e273. doi:10.1016/S2468-2667(22)00001-9

9. Mbivnjo EL, Lynch M, Huws JC. Measles outbreak investigation process in low- and middle-income countries: a systematic review of the methods and costs of contact tracing. Z Gesundh Wiss. 2022;30: 2407–2426. doi:10.1007/s10389-021-01590-2

10. Sagili KD, Muniyandi M, Shringarpure K, Singh K, Kirubakaran R, Rao R, et al. Strategies to detect and manage latent tuberculosis infection among household contacts of pulmonary TB patients in high TB burden countries - a systematic review and meta-analysis. Trop Med Int Health. 2022;27: 842–863. doi:10.1111/tmi.13808

11. Glennie M, Gardner K, Dowden M, Currie BJ. Active case detection methods for crusted scabies and leprosy: A systematic review. PLoS Negl Trop Dis. 2021;15: e0009577. doi:10.1371/journal.pntd.0009577

12. Kevin Jenniskens, Martin C J Bootsma, Johanna A A G Damen, Michiel S Oerbekke, Robin W M Vernooij, René Spijker, et al. Effectiveness of contact tracing apps for SARS-CoV-2: a rapid systematic review. BMJ Open. 2021;11: e050519. doi:10.1136/bmjopen-2021-050519

13. Thomas Craig KJ, Rizvi R, Willis VC, Kassler WJ, Jackson GP. Effectiveness of Contact Tracing for Viral Disease Mitigation and Suppression: Evidence-Based Review. JMIR Public Health Surveill. 2021;7: e32468. doi:10.2196/32468

14. Chung S-C, Marlow S, Tobias N, Alogna A, Alogna I, You S-L, et al. Lessons from countries implementing find, test, trace, isolation and support policies in the rapid response of the COVID-19 pandemic: a systematic review. BMJ Open. 2021;11: e047832. doi:10.1136/bmjopen-2020-047832

15. Mazza C, Girardi D, Gentile L, Gaeta M, Signorelli C, Odone A. Public health effectiveness of digital contact tracing in the COVID-19 pandemic: A systematic review of available data. Acta Biomed. 2021;92: e2021439. doi:10.23750/abm.v92iS6.12237

16. Velleca M, Malekinejad M, Miller C, Abascal Miguel L, Reeves H, Hopewell P, et al. The yield of tuberculosis contact investigation in low- and middle-income settings: a systematic review and meta-analysis. BMC Infect Dis. 2021;21: 1011. doi:10.1186/s12879-021-06609-3

17. Anglemyer A, Moore TH, Parker L, Chambers T, Grady A, Chiu K, et al. Digital contact tracing technologies in epidemics: a rapid review. Cochrane Database Syst Rev. 2020;2020: CD013699. doi:10.1002/14651858.CD013699

18. Taleghani S, Joseph-Davey D, West SB, Klausner HJ, Wynn A, Klausner JD. Acceptability and efficacy of partner notification for curable sexually transmitted infections in sub-Saharan Africa: A systematic review. Int J STD AIDS. 2019;30: 292–303. doi:10.1177/0956462418803983

19. Field E, Strathearn M, Boyd-Skinner C, Dyda A. Usefulness of linked data for infectious disease events: a systematic review. Epidemiol Infect. 2023;151: e46. doi:10.1017/S0950268823000316

20. Carter P, Megnin-Viggars O, Rubin GJ. What Factors Influence Symptom Reporting and Access to Healthcare During an Emerging Infectious Disease Outbreak? A Rapid Review of the Evidence. Health Security. 2021;19: 353–363. doi:10.1089/hs.2020.0126

21. Mathevet I, Ost K, Traverson L, Zinszer K, Ridde V. Accounting for health inequities in the design of contact tracing interventions: A rapid review. International Journal of Infectious Diseases. 2021;106: 65–70. doi:10.1016/j.ijid.2021.03.010

22. Megnin-Viggars O, Carter P, Melendez-Torres GJ, Weston D, Rubin GJ. Facilitators and barriers to engagement with contact tracing during infectious disease outbreaks: A rapid review of the evidence. PLoS One. 2020;15: e0241473. doi:10.1371/journal.pone.0241473

23. Amicosante AMV, Rosso A, Bernardini F, Guglielmi E, Eugeni E, Da Re F, et al. COVID-19 Contact Tracing Strategies During the First Wave of the Pandemic: Systematic Review of Published Studies. JMIR Public Health and Surveillance. 2023;9: e42678.

24. Oyibo K, Sahu KS, Oetomo A, Morita PP. Factors Influencing the Adoption of Contact Tracing Applications: Systematic Review and Recommendations. Front Digit Health. 2022;4: 862466. doi:10.3389/fdgth.2022.862466

25. Skarp JE, Downey LE, Ohrnberger JWE, Cilloni L, Hogan AB, Sykes AL, et al. A Systematic Review of the Costs Relating to Non-pharmaceutical Interventions Against Infectious Disease Outbreaks. Appl Health Econ Health Policy. 2021;19: 673–697. doi:10.1007/s40258-021-00659-z

26. Pegollo L, Maggioni E, Gaeta M, Odone A. Characteristics and determinants of population acceptance of COVID-19 digital contact tracing: a systematic review. Acta Biomed. 2021;92: e2021444. doi:10.23750/abm.v92iS6.12234

27. Akinbi A, Forshaw M, Blinkhorn V. Contact tracing apps for the COVID-19 pandemic: a systematic literature review of challenges and future directions for neo-liberal societies. Health Inf Sci Syst. 2021;9: 18. doi:10.1007/s13755-021-00147-7
