# Supplementary material for: Contact tracing strategies for infectious diseases: A systematic literature review
Source: PLOS Glob Public Health. 2025 May 9;5(5):e0004579. doi: 10.1371/journal.pgph.0004579 (PMC12063836; doi:10.1371/journal.pgph.0004579)
Supplement: S8 Table — (DOCX) [file pgph.0004579.s008.docx]

S8 Table. Comparative effectiveness of contact tracing strategies

| **Author year** | **Geography** | **Disease** | **Intervention** | **Comparator** | **Outcome** |
| --- | --- | --- | --- | --- | --- |
| **Golden *et al.* 2007**[1] | United States | Gonorrhea/ chlamydia | Partner notification program with promoting Patient-Delivered Partner Therapy (PDPT) use and providing free medications to clinicians for use as PDPT | Before application of program | The percentage of cases who received PDPT increased (16% vs 5.6%) and cases whose partners were all treated with a program increased (65% vs 39%) |
| **Myint *et al.* 2019**[2] | Ayeyarwady Region, Myanmar | Tuberculosis (TB) | Traditional CT (Staff visiting the households of Index cases) + Telephone call by program nurse | Traditional CT | The TB Yield Among Screened Household Contacts increased (6.4% vs 0.3%) |
| **Ospina *et al.* 2012**[3] | Spain, Barcelona | TB | Community Health Worker (CHW) involved in CT | No involvement | The proportion of cases increased significantly for whom CT is completed (81.6% vs 65.7%, p <0.001) |
| **Charbonnier *et al.* 2021**[4] | France | COVID-19 | Home visits by nurses for screening close contacts for SARS-CoV-2 by Reverse Transcriptase- Polymerase Chain Reaction (RT-PCR) and serological Rapid Diagnostic Test (RDT) | Only using RT-PCR | The proportion of contacts isolated decreased to 73% in the group tested with RT-PCR/RDT, compared to 100% in the group tested with RT-PCR alone. |
| **Estcourt *et al.* 2015**[5] | England, London | Chlamydia | APTPharmacy, APTHotline | Standard Partner Notification (PN) | Time to Partner Treatment was median of 0 day (IQR 0 – 0) for APTHotline and Routine PN groups and median of 0 day (IQR 0 – 4) for APTPharmacy group |
| **Evans *et al.* 2022**[6] | United States | COVID-19 | CT interview with memory retrieval techniques | CT interview without memory retrieval techniques | The mean number of close contacts reported under the enhanced protocol was 51% higher than that reported under the standard protocol (12.14 vs 8.01). |
| **Fatima *et al.* 2016**[7] | Pakistan | TB | CT within a radius of 50 meters from the household of index patient | Before applying the limit of 50 meters | After implementing the limit, there was a 7.9% increase in TB detection cases compared to the period before the limit was applied. |
| **Beebeejaun *et al.* 2021**[8] | England | Hepatitis B Virus (HBV) | Nurse-led enhanced CT and management of Chronic Hepatitis B (CHB) | No nurse-led enhanced CT and management of CHB | Following the implementation of the intervention, there was a significant increase in the referral rate of contacts to a specialist, rising from 86% to 99.7% (p<0.001). Additionally, the rate of testing among close contacts escalated from 34% to 94%, and the vaccination rate improved from 77% to 93%. |
| **Österlund *et al.* 2005**[9] | Sweden | Chlamydia | CT with ompA genotyping with specialized midwives | CT without genotyping or specialized midwives | Higher contacts identified after implementing the genotyping and specialized midwives (2.1 vs 1.6). |
| **Rekha *et al.* 2013**[10] | India | TB | After Isoniazid Preventive Therapy (IPT) register and card of Revised National Tuberculosis Control Program | Before the intervention | Post-intervention, the coverage of IPT expanded significantly from 19% to 61%, with OR of 6.63 (95% CI 3.14-14.14, p <0.001). |
| **Duarte *et al.* 2012**[11] | Portugal | TB | CT screening of close contacts through interview and home & workplace visits | CT screening of close contacts through interview only | Post-intervention, the proportion of screened contacts increased from 67.6% to 87.3% and the rate of at-risk contacts identification increased from 2.5 to 8.4 per case. The number of prevented cases of active TB increased from 5 to 10. And the treatment completion rate increased from 83% to 96%. |
| **Salazar-Austin *et al.* 2020**[12] | South Africa | TB | CT using symptom screening | Tuberculin Skin Test (TST)-based CT screening | Symptom screening resulted in a lower proportion of identified contacts starting treatment (51.5% vs 57.1%). Completion rates of 6-month tuberculosis preventive treatment (TPT) for child contacts were comparable between groups (14% vs 15%, p = 0.89). There were modest increases in the adjusted proportion of estimated child contacts identified (55% vs 45%, p = 0.27) and those screened (30% vs 27%; p = 0.39). |
| **Lu *et al.* 2023**[13] | US | COVID-19 | High-touch CT (integrated with social services) | Standard CT | High-touch CT significantly increased the referral rate by 2.92% (95% CI: 0.3 - 5.53, p<0.05). The uptake rate increased by 1.7% (95% CI: 0.7 - 5.53, p = 0.06) |
| **Edmiston *et al.* 2010**[14] | Australia | Chlamydia | Clients advised about the need for contact tracing and were offered letters for contacts’ doctors + ‘Make Contact’ cards | No ‘Make contact’ cards | There were no significant differences between the groups regarding the mean number of contacts notified (1.83 vs 1.91, p = 0.74) and the mean number of contacts treated (0.94 vs 0.91, p = 0.89). |
| **Weaver *et al.* 2022**[15] | United States | COVID-19 | CT in the Emergency Department (ED) | Standard CT | CT in the ED achieved higher mean contacts identified per case (2.8 vs 1.4) and higher contacts reached out (83% vs 78%, p = 0.16) |
| **Ansari *et al.* 1998**[16] | Wales | TB | The strategy included identifying TB patients and their contacts, recording specific details about both, and comparing the screening procedure followed with the local protocol | Previous protocol | The contact screening rate was the same between the two protocols (96.5% vs 97.5%). The new strategy, compared to the previous one, decreased unnecessary screening rates (14.4% vs 23.3%) and improved testing rates (Heaf tests: 89% vs 55.7%; chest radiographs: 63% vs 33.9%). The Bacillus Calmette-Guérin (BCG) vaccination rate increased to 22.8% from 20% under the previous protocol. |
| **Davis *et al.* 2019**[17] | Uganda | TB | CHWs first offered home Human Immunodeficiency Virus (HIV) counselling and testing to contacts and category-specific messages reporting results and/or follow-up instructions | CHWs referred contacts with indications for TB testing and/or clinical evaluation to health facilities for standard sputum examination, HIV counselling and testing | The median number of contacts reported per case was identical between the two strategies (3 (IQR: 1–4) vs 3 (IQR: 2–4)). The proportion of household contacts completing TB evaluation within 14 days was comparable (14% vs 15%). However, the intervention group had a higher rate of contacts diagnosed with microbiologically confirmed TB (1.5% vs 1.1%). |

Abbreviations: APT: Accelerated partner therapy; BCG: Bacillus Calmette-Guérin; CHW: Community Health Worker; CI: confidence interval; CT: contact tracing; ED: emergency department; HIV: Human Immunodeficiency Virus; IPT: Isoniazid Preventive Therapy; OR: odds ratio; PDPT: Patient-Delivered Partner Therapy; IQR: interquartile range; RDT: rapid diagnostic test; RT-PCR: reverse transcriptase-polymerase chain reaction; SARS-CoV-2: severe acute respiratory syndrome coronavirus 2; TB: tuberculosis; TPT: tuberculosis preventive treatment; TST: Tuberculin Skin Test.

1. Golden MR, Hughes JP, Brewer DD, Holmes KK, Whittington WLH, Hogben M, et al. Evaluation of a population-based program of expedited partner therapy for gonorrhea and chlamydial infection. Sex Transm Dis. 2007;34: 598–603. doi:10.1097/01.olq.0000258319.54316.06

2. Myint O, Sriplung H, San CC, Chongsuvivatwong V. Additional active tuberculosis cases detected and costs incurred by a second household contact investigation. Public Health Action. 2019;9: 182. doi:10.5588/pha.19.0028

3. Ospina JE, Orcau À, Millet J-P, Sánchez F, Casals M, Caylà JA. Community health workers improve contact tracing among immigrants with tuberculosis in Barcelona. BMC Public Health. 2012;12: 158. doi:10.1186/1471-2458-12-158

4. Charbonnier L, Rouprêt-Serzec J, Caseris M, Danse M, Cointe A, Cohen L, et al. Contribution of Serological Rapid Diagnostic Tests to the Strategy of Contact Tracing in Households Following SARS-CoV-2 Infection Diagnosis in Children. Front Pediatr. 2021;9: 638502. doi:10.3389/fped.2021.638502

5. Estcourt CS, Sutcliffe LJ, Copas A, Mercer CH, Roberts TE, Jackson LJ, et al. Developing and testing accelerated partner therapy for partner notification for people with genital Chlamydia trachomatis diagnosed in primary care: a pilot randomised controlled trial. Sex Transm Infect. 2015;91: 548–554. doi:10.1136/sextrans-2014-051994

6. Evans JR, Dawson HR, Chae H, Goldfarb D, Fisher RP, Dianiska RE, et al. Enhancing the effectiveness of contact tracing interviews: A randomized controlled experiment of an enhanced cognitive interview protocol. Am J Infect Control. 2022;50: 631–637. doi:10.1016/j.ajic.2021.12.015

7. Fatima R, Qadeer E, Yaqoob A, Haq MU, Majumdar SS, Shewade HD, et al. Extending “Contact Tracing” into the Community within a 50-Metre Radius of an Index Tuberculosis Patient Using Xpert MTB/RIF in Urban, Pakistan: Did It Increase Case Detection? PLoS One. 2016;11: e0165813. doi:10.1371/journal.pone.0165813

8. Beebeejaun K, Amin-Chowdhury Z, Letley L, Kara E, Mahange B, Harrington K, et al. Impact of a nurse-led enhanced monitoring, management and contact tracing intervention for chronic hepatitis B in England, 2015-2017. J Viral Hepat. 2021;28: 72–79. doi:10.1111/jvh.13403

9. Osterlund A, Persson T, Persson I, Lysén M, Herrmann B. Improved contact tracing of Chlamydia trachomatis in a Swedish county--is genotyping worthwhile? Int J STD AIDS. 2005;16: 9–13. doi:10.1258/0956462052932809

10. Rekha B, Jagarajamma K, Chandrasekaran V, Wares F, Sivanandham R, Swaminathan S. Improving screening and chemoprophylaxis among child contacts in India’s RNTCP: a pilot study. Int J Tuberc Lung Dis. 2013;17: 163–168. doi:10.5588/ijtld.12.0415

11. Duarte R, Neto M, Carvalho A, Barros H. Improving tuberculosis contact tracing: the role of evaluations in the home and workplace. Int J Tuberc Lung Dis. 2012;16: 55–59. doi:10.5588/ijtld.10.0511

12. Salazar-Austin N, Cohn S, Barnes GL, Tladi M, Motlhaoleng K, Swanepoel C, et al. Improving Tuberculosis Preventive Therapy Uptake: A Cluster-randomized Trial of Symptom-based Versus Tuberculin Skin Test-based Screening of Household Tuberculosis Contacts Less Than 5 Years of Age. Clin Infect Dis. 2020;70: 1725–1732. doi:10.1093/cid/ciz436

13. Lu LC, Ouyang D, D’Agostino A, Diaz A, Rudman SL, Ho DE. Integrating social services with disease investigation: A randomized trial of COVID-19 high-touch contact tracing. PLoS One. 2023;18: e0285752. doi:10.1371/journal.pone.0285752

14. Edmiston N, Merritt T, Ooi C. Make contact: a comparative study of contact tracing strategies. Int J STD AIDS. 2010;21: 431–434. doi:10.1258/ijsa.2010.010118

15. Weaver SC, Byrne SS, Bruce H, Vargas OL, Robey TE. Prospective Case-control Study of Contact Tracing Speed for Emergency Department-based Contact Tracers. West J Emerg Med. 2022;23: 623–627. doi:10.5811/westjem.2022.5.53196

16. Ansari S, Thomas S, Campbell IA, Furness L, Evans MR. Refined tuberculosis contact tracing in a low incidence area. Respir Med. 1998;92: 1127–1131. doi:10.1016/s0954-6111(98)90406-1

17. Davis JL, Turimumahoro P, Meyer AJ, Ayakaka I, Ochom E, Ggita J, et al. Home-based tuberculosis contact investigation in Uganda: a household randomised trial. ERJ Open Res. 2019;5: 00112–02019. doi:10.1183/23120541.00112-2019
