# Supplementary figures and images for: Contact tracing strategies for infectious diseases: A systematic literature review
Source: PLOS Glob Public Health. 2025 May 9;5(5):e0004579. doi: 10.1371/journal.pgph.0004579 (PMC12063836; doi:10.1371/journal.pgph.0004579)

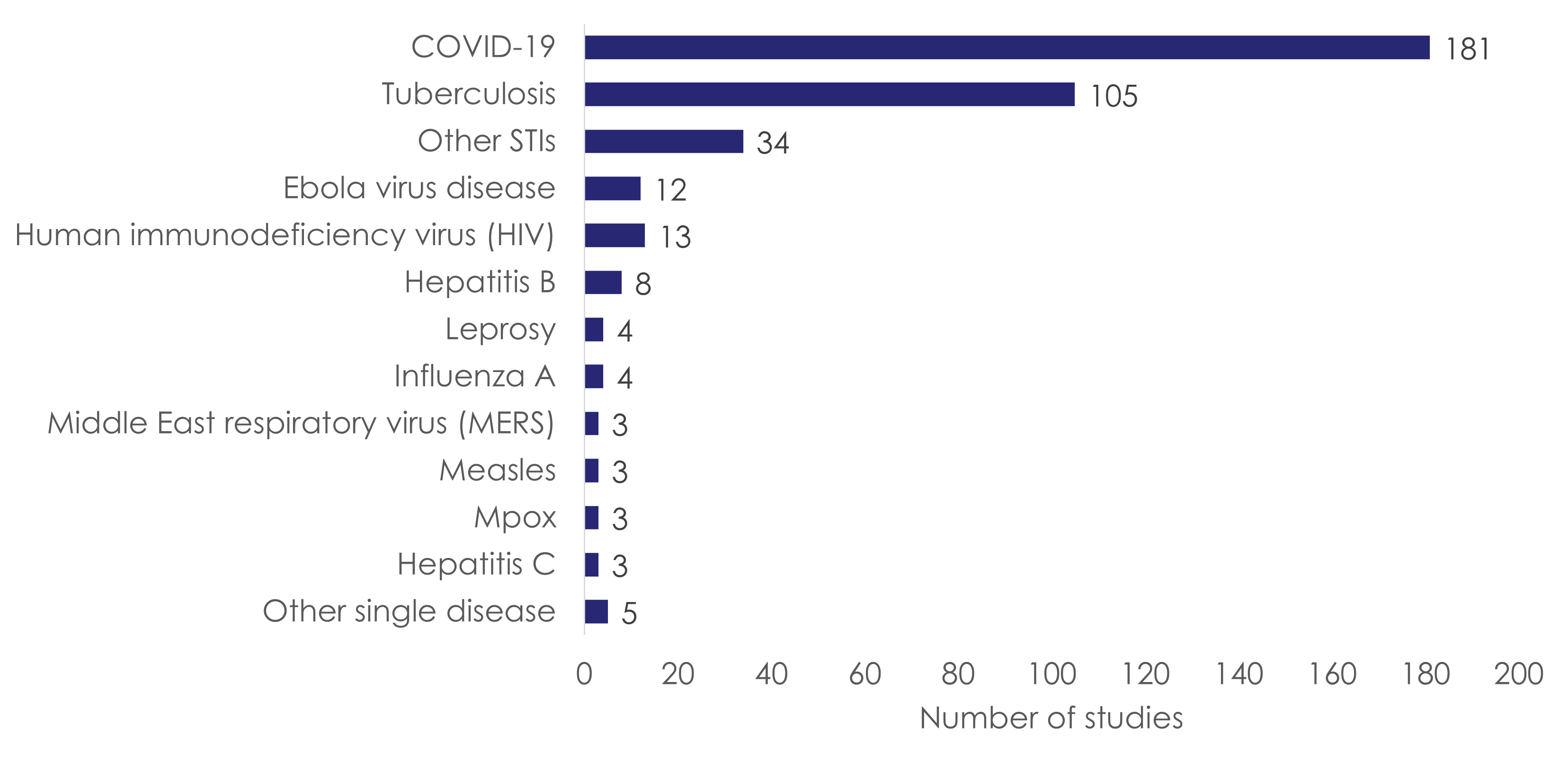

Supplement: S1 Fig — Note: Some studies considered more than one disease. “Other single disease” includes methicillin-resistant Staphylococcus aureus (MRSA) infection, mumps, pertussis, severe acute respiratory syndrome (SARS), scarlet fever, (n = 1 for each). (TIF) [file pgph.0004579.s009.tif]

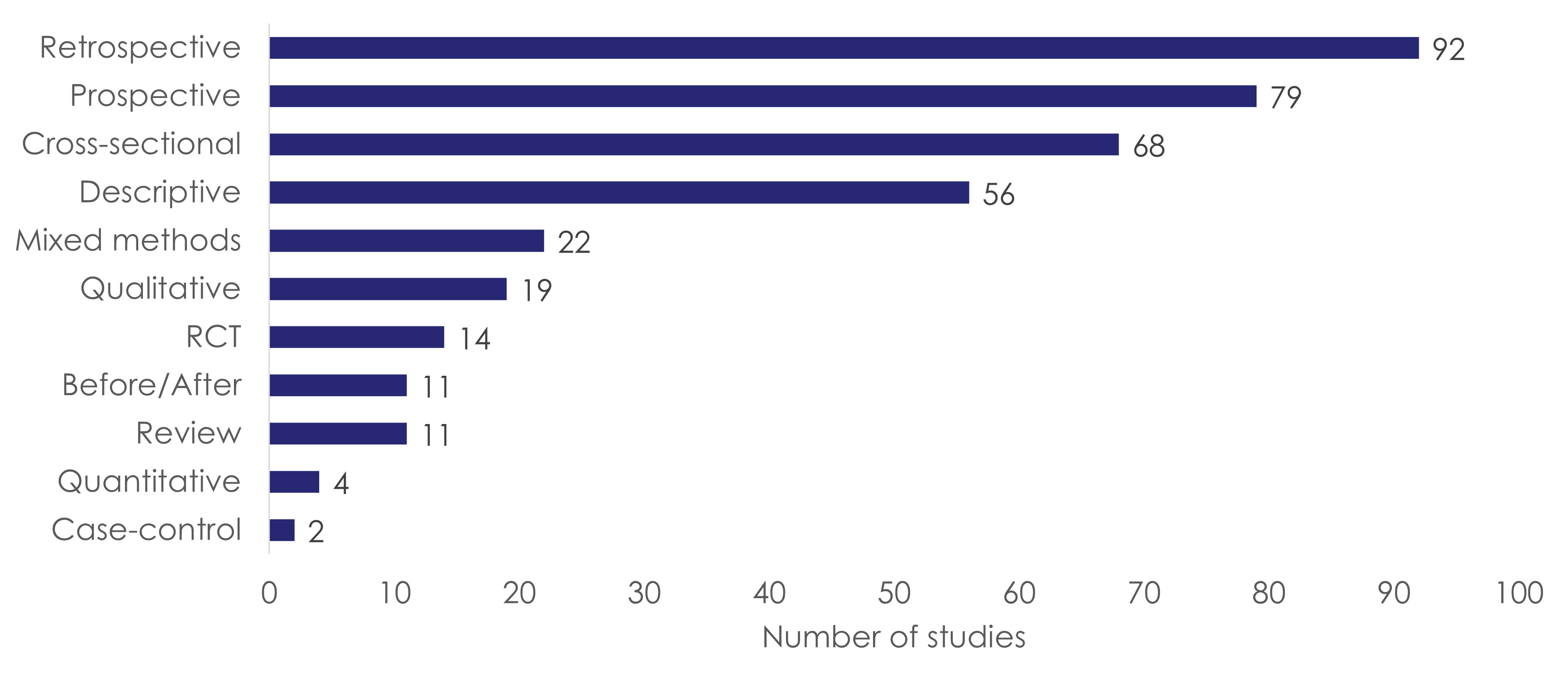

Supplement: S2 Fig — (TIF) [file pgph.0004579.s010.tif]

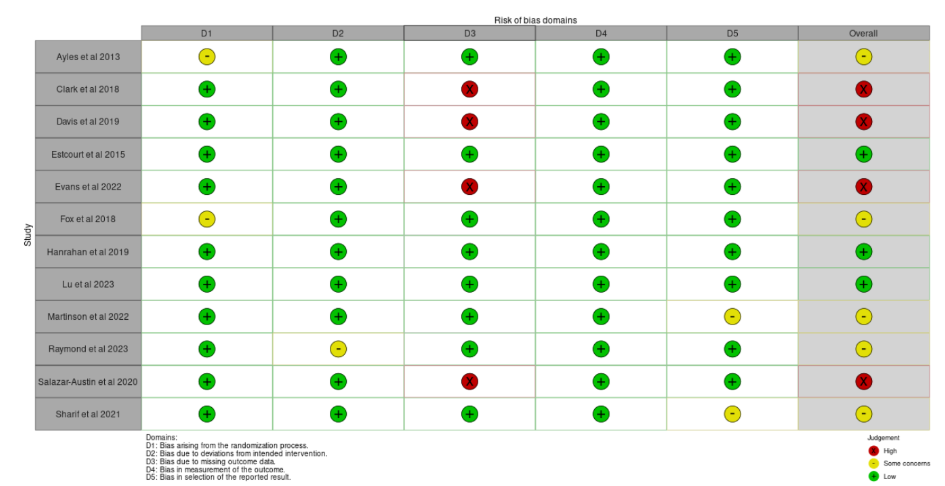

Supplement: S3 Fig — (PNG) [file pgph.0004579.s011.png]

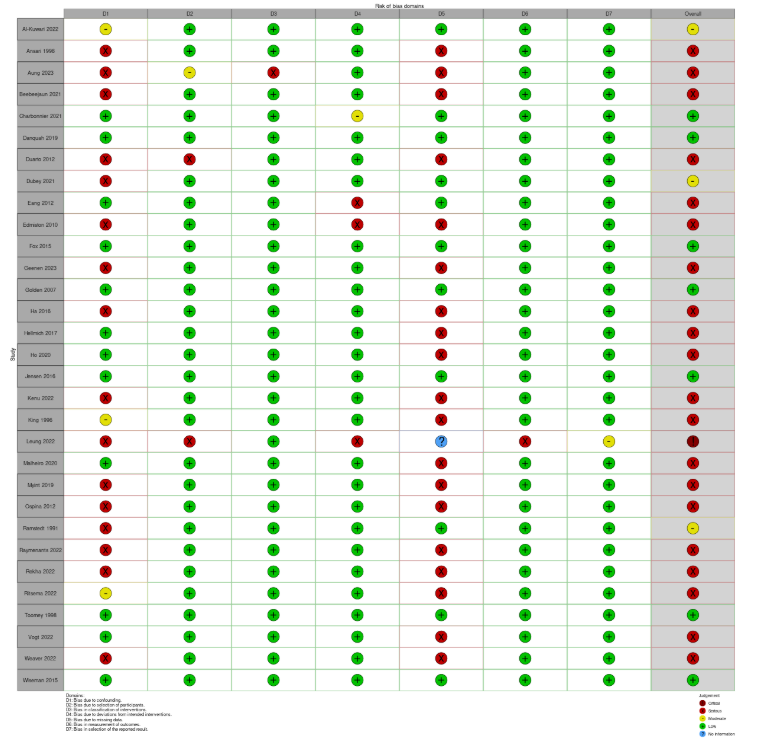

Supplement: S4 Fig — (PNG) [file pgph.0004579.s012.png]

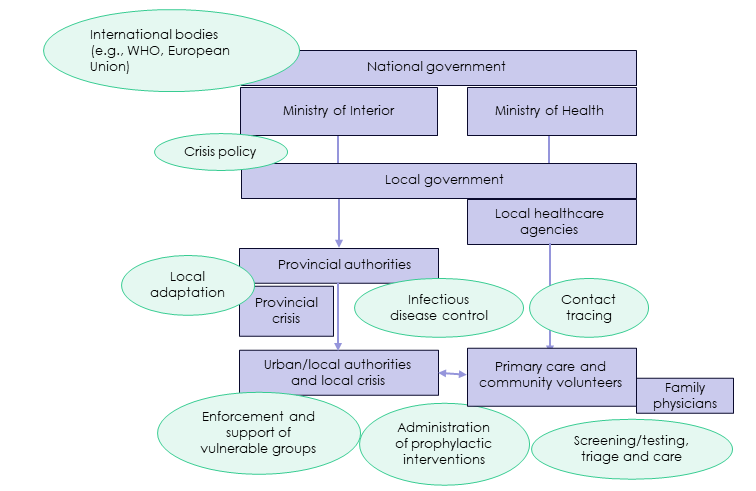

Supplement: S5 Fig — (TIF) [file pgph.0004579.s013.tif]
