# Supplementary material for: Contact tracing strategies for infectious diseases: A systematic literature review
Source: PLOS Glob Public Health. 2025 May 9;5(5):e0004579. doi: 10.1371/journal.pgph.0004579 (PMC12063836; doi:10.1371/journal.pgph.0004579)
Supplement: S1 Box — (DOCX) [file pgph.0004579.s014.docx]

S1 Box. Key definitions and elements of contact tracing strategies

### Contact person definition

Most studies examining contact tracing for COVID-19 and one mumps defined contact persons based on **physical proximity**, specifically in COVID-19 studies within 1.5 to two meters (five to six feet) of a confirmed case with an exposure of at least 15 minutes.[1]

Studies on TB most often defined those individuals as contact persons who were within an index case’s **community or shared settings and had significant interaction** with the index case. [2–22] A study on pertussis added to this definition by including all individuals that had face-to-face contact with the index case.[23]

In Ebola research, contact person definitions prioritized **direct physical interactions** with symptomatic patients or their bodily fluids, indicating a consistent high risk of transmission through such encounters. Chung *et al.* (2015) outlined this by including criteria like ‘being within 90 cm or three feet for over 15 minutes in their contact definition’. [24]

For STIs, contact person definitions were primarily framed around **sexual partnerships and their timing**. Studies predominantly identified sexual partners, whether casual or regular, as contacts.[25–31] Manavi *et al.* provided a nuanced view by differentiating between casual and regular partnerships, suggesting that the type of sexual relationship influences the likelihood of disease transmission and the subsequent need for contact tracing.[25]

Studies focusing on Hepatitis B and C identified contact persons through behaviors associated with transmission risks, such as **sharing drug injection equipment** or injecting drugs in proximity.[32–34]

### High-risk contact persons

Studies defining high-risk contact persons most often made no differentiation in identifying a person at high risk of infection due to proximity to an infected case or the individual’s health status, thus using ‘close contact’ and ‘high-risk contact’ interchangeably.

COVID-19 studies frequently defined both close contacts and high-risk contacts as those in close proximity (less than 1.5 or two meters) and extended duration (at least 15

minutes) or face-to-face contact to a case without Personal Protective Equipment (PPE). TB studies often defined close and high-risk contacts as those exposed to a case for an extended period of time (e.g. several hours per day,[35] or for more than an hour several times per week[36]) or, in closed and poorly ventilated rooms without respiratory protection.[36–38] Ebola and mpox studies similarly defined close and high-risk contacts as those with unprotected direct contact to a case or their bodily fluids.[2,39–41]

Notably, two TB studies did make a differentiation between close and high-risk contacts. Bagdasarian *et al.*[42] and Park *et al.*[37] focused on the vulnerability of contacts, particularly those who are immunocompromised or have conditions that increase their susceptibility to infection. This additional definition of a ‘high-risk contact’ is crucial as it underscores the need to identify and protect individuals who are susceptible to severe disease outcomes upon exposure.[37,42]

1. Bharti N, Exten C, Fulton V, Oliver-Veronesi R. Lessons from managing a campus mumps outbreak using test, trace, and isolate efforts. Am J Infect Control. 2021;49: 849–851. doi:10.1016/j.ajic.2020.11.008

2. Hoang TTT, Nguyen VN, Dinh NS, Thwaites G, Nguyen TA, van Doorn HR, et al. Active contact tracing beyond the household in multidrug resistant tuberculosis in Vietnam: a cohort study. BMC Public Health. 2019;19: 241. doi:10.1186/s12889-019-6573-z

3. Mandalakas AM, Ngo K, Alonso Ustero P, Golin R, Anabwani F, Mzileni B, et al. BUTIMBA: Intensifying the Hunt for Child TB in Swaziland through Household Contact Tracing. PLoS One. 2017;12: e0169769. doi:10.1371/journal.pone.0169769

4. Kadyrov M, Thekkur P, Geliukh E, Sargsyan A, Goncharova O, Kulzhabaeva A, et al. Contact Tracing and Tuberculosis Preventive Therapy for Household Child Contacts of Pulmonary Tuberculosis Patients in the Kyrgyz Republic: How Well Are We Doing? Trop Med Infect Dis. 2023;8: 332. doi:10.3390/tropicalmed8070332

5. Imsanguan W, Bupachat S, Wanchaithanawong V, Luangjina S, Thawtheong S, Nedsuwan S, et al. Contact tracing for tuberculosis, Thailand. Bull World Health Organ. 2020;98: 212–218. doi:10.2471/BLT.19.239293

6. Tefera F, Barnabee G, Sharma A, Feleke B, Atnafu D, Haymanot N, et al. Evaluation of facility and community-based active household tuberculosis contact investigation in Ethiopia: a cross-sectional study. BMC Health Serv Res. 2019;19: 234. doi:10.1186/s12913-019-4074-5

7. Moyo N, Tay EL, Denholm JT. Evaluation of tuberculin skin testing in tuberculosis contacts in Victoria, Australia, 2005-2013. Public Health Action. 2015;5: 188–193. doi:10.5588/pha.15.0018

8. Fatima R, Qadeer E, Yaqoob A, Haq MU, Majumdar SS, Shewade HD, et al. Extending “Contact Tracing” into the Community within a 50-Metre Radius of an Index Tuberculosis Patient Using Xpert MTB/RIF in Urban, Pakistan: Did It Increase Case Detection? PLoS One. 2016;11: e0165813. doi:10.1371/journal.pone.0165813

9. Tlale L, Frasso R, Kgosiesele O, Selemogo M, Mothei Q, Habte D, et al. Factors influencing health care workers’ implementation of tuberculosis contact tracing in Kweneng, Botswana. Pan Afr Med J. 2016;24: 229. doi:10.11604/pamj.2016.24.229.7004

10. Eang MT, Satha P, Yadav RP, Morishita F, Nishikiori N, van-Maaren P, et al. Early detection of tuberculosis through community-based active case finding in Cambodia. BMC Public Health. 2012;12: 469. doi:10.1186/1471-2458-12-469

11. Chakhaia T, Magee MJ, Kempker RR, Gegia M, Goginashvili L, Nanava U, et al. High utility of contact investigation for latent and active tuberculosis case detection among the contacts: a retrospective cohort study in Tbilisi, Georgia, 2010-2011. PLoS One. 2014;9: e111773. doi:10.1371/journal.pone.0111773

12. Izumi K, Ohkado A, Uchimura K, Kawatsu L, Suenaga M, Urakawa M, et al. Evaluation of tuberculosis contact investigations in Japan. Int J Tuberc Lung Dis. 2017;21: 188–195. doi:10.5588/ijtld.16.0508

13. Koster B, Borgen K, Meijer H, Plas S van der, Kuyvenhoven V. Large scale contact tracing after a case of open tuberculosis in a supermarket, the Netherlands, January - February 2005. Weekly releases (1997–2007). 2005;10: 2648. doi:10.2807/esw.10.08.02648-en

14. Ansari S, Thomas S, Campbell IA, Furness L, Evans MR. Refined tuberculosis contact tracing in a low incidence area. Respir Med. 1998;92: 1127–1131. doi:10.1016/s0954-6111(98)90406-1

15. Arnold A, Witney AA, Vergnano S, Roche A, Cosgrove CA, Houston A, et al. XDR-TB transmission in London: Case management and contact tracing investigation assisted by early whole genome sequencing. J Infect. 2016;73: 210–218. doi:10.1016/j.jinf.2016.04.037

16. Cavany SM, Vynnycky E, Sumner T, Macdonald N, Thomas HL, White J, et al. Transmission events revealed in tuberculosis contact investigations in London. Sci Rep. 2018;8: 6676. doi:10.1038/s41598-018-25149-6

17. Mwansa-Kambafwile J, McCarthy K, Gharbaharan V, Venter FWD, Maitshotlo B, Black A. Tuberculosis case finding: evaluation of a paper slip method to trace contacts. PLoS One. 2013;8: e75757. doi:10.1371/journal.pone.0075757

18. Banner P. Tuberculosis contact tracing within a school environment: lessons for the future. N S W Public Health Bull. 2013;24: 27–28. doi:10.1071/NB12096

19. Martin-Sanchez M, Brugueras S, de Andrés A, Simon P, Gorrindo P, Ros M, et al. Tuberculosis incidence among infected contacts detected through contact tracing of smear-positive patients. PLoS One. 2019;14: e0215322. doi:10.1371/journal.pone.0215322

20. Gashu Z, Jerene D, Ensermu M, Habte D, Melese M, Hiruy N, et al. The Yield of Community-Based “Retrospective” Tuberculosis Contact Investigation in a High Burden Setting in Ethiopia. PLoS One. 2016;11: e0160514. doi:10.1371/journal.pone.0160514

21. Kigozi NG, Heunis JC, Engelbrecht MC. Yield of systematic household contact investigation for tuberculosis in a high-burden metropolitan district of South Africa. BMC Public Health. 2019;19: 867. doi:10.1186/s12889-019-7194-2

22. S K, H M, M H, A S, Mr M. Transmission of Mycoba cterium tuberculosis to households of tuberculosis patients: a comprehensive contact tracing study. Archives of Iranian medicine. 2006;9. Available: https://pubmed.ncbi.nlm.nih.gov/16859052/

23. Hellmich TR, Clements CM, El-Sherif N, Pasupathy KS, Nestler DM, Boggust A, et al. Contact tracing with a real-time location system: A case study of increasing relative effectiveness in an emergency department. Am J Infect Control. 2017;45: 1308–1311. doi:10.1016/j.ajic.2017.08.014

24. Chung WM, Smith JC, Weil LM, Hughes SM, Joyner SN, Hall EM, et al. Active Tracing and Monitoring of Contacts Associated With the First Cluster of Ebola in the United States. Ann Intern Med. 2015;163: 164–173. doi:10.7326/M15-0968

25. Manavi K, Bhaduri S, Tariq A, West Midlands British Association of Sexual Health Audit Group. Audit on the success of partner notification for sexually transmitted infections in the West Midlands. Int J STD AIDS. 2008;19: 856–858. doi:10.1258/ijsa.2008.008130

26. Wu X, Hong F, Zhang C, Feng T, Lan L, Yang Y. [Contact tracing of pregnant women infected with syphilis and the associated factors]. Zhonghua Yu Fang Yi Xue Za Zhi. 2015;49: 1067–1072.

27. Yan R, Deng B, Wen G, Huang L, Li L, Huang Z. Contact tracing of syphilis-seropositive pregnant women and syphilis-infection among their male partners in Bao’an district, Shenzhen, China. BMC Infect Dis. 2020;20: 684. doi:10.1186/s12879-020-05403-x

28. Cope AB, Bernstein KT, Matthias J, Rahman M, Diesel JC, Pugsley RA, et al. Effectiveness of Syphilis Partner Notification After Adjusting for Treatment Dates, 7 Jurisdictions. Sex Transm Dis. 2022;49: 160–165. doi:10.1097/OLQ.0000000000001518

29. Oh MK, Boker JR, Genuardi FJ, Cloud GA, Reynolds J, Hodgens JB. Sexual contact tracing outcome in adolescent chlamydial and gonococcal cervicitis cases. J Adolesc Health. 1996;18: 4–9. doi:10.1016/1054-139X(95)00109-6

30. Winfield J, Latif AS. Tracing contacts of persons with sexually transmitted diseases in a developing country. Sex Transm Dis. 1985;12: 5–7. doi:10.1097/00007435-198501000-00002

31. Clark JL, Segura ER, Oldenburg CE, Salvatierra HJ, Rios J, Perez-Brumer AG, et al. Traditional and Web-Based Technologies to Improve Partner Notification Following Syphilis Diagnosis Among Men Who Have Sex With Men in Lima, Peru: Pilot Randomized Controlled Trial. J Med Internet Res. 2018;20: e232. doi:10.2196/jmir.9821

32. Götz HM, van Doornum G, Niesters HG, den Hollander JG, Thio HB, de Zwart O. A cluster of acute hepatitis C virus infection among men who have sex with men--results from contact tracing and public health implications. AIDS. 2005;19: 969–974. doi:10.1097/01.aids.0000171412.61360.f8

33. Eckhardt B, Aponte-Melendez Y, Kapadia SN, Mateu-Gelabert P. Contact tracing in acute hepatitis C: The source patient identification and group overlap therapy proof-of-concept pilot program. Clin Liver Dis (Hoboken). 2022;20: 72–76. doi:10.1002/cld.1242

34. Brewer DD, Hagan H. Evaluation of a patient referral contact tracing programme for hepatitis B and C virus infection in drug injectors. Euro Surveill. 2009;14: 5–9.

35. del Castillo Otero D, Peñafiel Colás M, Alvarez Gutiérrez F, Soto Campos JG, Calderón Osuna E, Toral Marín J, et al. Investigation of tuberculosis contacts in a nonhospital pneumology practice. Eur J Clin Microbiol Infect Dis. 1999;18: 790–795. doi:10.1007/s100960050402

36. Corbett C, Kulzhabaeva A, Toichkina T, Kalmambetova G, Ahmedov S, Antonenka U, et al. Implementing contact tracing for tuberculosis in Kyrgyz Republic and risk factors for positivity using QuantiFERON-TB Gold plus. BMC Infect Dis. 2020;20: 746. doi:10.1186/s12879-020-05465-x

37. Park SY, Lee EJ, Kim YK, Lee SY, Kim GE, Jeong YS, et al. Aggressive Contact Investigation of In-Hospital Exposure to Active Pulmonary Tuberculosis. J Korean Med Sci. 2019;34: e58. doi:10.3346/jkms.2019.34.e58

38. André E, Rusumba O, Evans CA, Ngongo P, Sanduku P, Elvis MM, et al. Patient-led active tuberculosis case-finding in the Democratic Republic of the Congo. Bull World Health Organ. 2018;96: 522–530. doi:10.2471/BLT.17.203968

39. Senga M, Koi A, Moses L, Wauquier N, Barboza P, Fernandez-Garcia MD, et al. Contact tracing performance during the Ebola virus disease outbreak in Kenema district, Sierra Leone. Philos Trans R Soc Lond B Biol Sci. 2017;372: 20160300. doi:10.1098/rstb.2016.0300

40. Santos A, Nuñez G, La S, Dorsey JH, Patel SS, Luk KG, et al. Digital Point Solutions for Extending Contact Tracing Capacity. Sex Transm Dis. 2023;50: S41–S47. doi:10.1097/OLQ.0000000000001712

41. Wolfe CM, Hamblion EL, Schulte J, Williams P, Koryon A, Enders J, et al. Ebola virus disease contact tracing activities, lessons learned and best practices during the Duport Road outbreak in Monrovia, Liberia, November 2015. PLoS Negl Trop Dis. 2017;11: e0005597. doi:10.1371/journal.pntd.0005597

42. Bagdasarian N, Chan HC, Ang S, Isa MS, Chan SM, Fisher DA. A “Stone in the Pond” Approach to Contact Tracing: Responding to a Large-Scale, Nosocomial Tuberculosis Exposure in a Moderate TB-Burden Setting. Infect Control Hosp Epidemiol. 2017;38: 1509–1511. doi:10.1017/ice.2017.228
